# A Notch-mediated, temporal asymmetry in BMP pathway activation promotes photoreceptor subtype diversification

**DOI:** 10.1371/journal.pbio.2006250

**Published:** 2019-01-31

**Authors:** Elise Cau, Brice Ronsin, Laurianne Bessière, Patrick Blader

**Affiliations:** Centre de Biologie du Développement (Unité Mixte de Recherche 5547), Centre de Biologie Intégrative (Fédération de Recherche 3743), Université de Toulouse, Centre National de la Recherche Scientifique, Université Paul Sabatier, Toulouse, France; New York University, United States of America

## Abstract

Neural progenitors produce neurons whose identities can vary as a function of the time that specification occurs. Here, we describe the heterochronic specification of two photoreceptor (PhR) subtypes in the zebrafish pineal gland. We find that accelerating PhR specification by impairing Notch signaling favors the early fate at the expense of the later fate. Using in vivo lineage tracing, we show that most pineal PhRs are born from a fate-restricted progenitor. Furthermore, sister cells derived from the division of PhR-restricted progenitors activate the bone morphogenetic protein (BMP) signaling pathway at different times after division, and this heterochrony requires Notch activity. Finally, we demonstrate that PhR identity is established as a function of when the BMP pathway is activated. We propose a novel model in which division of a progenitor with restricted potential generates sister cells with distinct identities via a temporal asymmetry in the activation of a signaling pathway.

## Author summary

A major goal in the field of developmental neurobiology is to identify the mechanisms that underly the diversification of the subtypes of neurons that are needed for the function of the nervous system. When investigating these mechanisms, time is an often-overlooked variable. Here, we show that in the zebrafish pineal gland—a neuroendocrine organ containing mostly photoreceptors (PhRs) and projection neurons—different classes of PhRs appear in a temporal sequence. In this simple system, the decision to adopt a PhR fate is driven by the activation of the bone morphogenetic protein (BMP) signaling pathway. Following the final cell division of a PhR progenitor, the sister cells normally activate the BMP pathway at different times. When Notch signaling activity is abrogated, activation of the BMP pathway occurs earlier and synchronously, which in turn favors the development of early PhR fates at the expense of later fates. We propose a model in which preventing sister cells from activating a signaling pathway in a synchronous fashion after their final division allows diversification of cell fates.

## Introduction

The development of a functional nervous system requires the production of an amazing diversity of cell types. The precise identity of each neuron is acquired through a complex process referred to as neuronal subtype specification. Although different molecular mechanisms have been reported to control the specification of neuronal subtype identity, the activity of signaling pathways is at the heart of this process. Bone morphogenetic proteins (BMPs) have been widely linked with neuronal specification, such as in the mouse retina, where they promote the expression of M-opsin at the expense of S-opsin in photoreceptors (PhRs) [1,2]. Cell–cell communication involving the Notch pathway has also been implicated in neural specification [3]. In numerous cases, neuronal subtype specification is influenced by the concomitant activity of several signaling pathways, but the mechanisms underlying how these signals collaborate to establish distinct neural subtypes are only now beginning to be uncovered [4–7]. For instance, progenitors in the p2 domain of the spinal cord have the choice between the v2a and the v2b interneuron fate. In this system, BMP and Notch cooperate to promote the v2b fate [8–11], with Notch acting to promote activation of the BMP pathway in the future v2b cell [5]. These two pathways are also involved in neural subtype specification in the zebrafish pineal gland but with the roles being reversed [6]; BMP operates first to promote responsiveness to Notch signaling during the choice between PhR and projection neuron (PN) fates.

Neuronal subtype specification can also be temporally guided, with different neuronal fates being produced over time from a common pool of progenitors. Indeed, it has been shown that neuronal progenitors in the vertebrate retina and spinal cord, as well as the mammalian cortex and olfactory bulb, generate distinct subtypes of neurons depending on when they are produced. In one model, the sequential production of distinct neuronal subtypes is the result of the evolution in the competence of neuronal progenitors [12]. In invertebrates, feed-forward cascades of temporal transcription factors have been described that control the evolution of competence within neural progenitors (see [13] for a review). Although this mechanism has apparently been conserved in vertebrates, so far only three factors have been identified that promote early or late fates; whereas forkhead box G1 (Foxg1) suppresses and IKAROS family zinc finger 1 (Ikzf1) promotes early cortical fates [14,15], Ikzf1 promotes early fates and castor zinc finger 1 (Casz1) promotes late fates in the retina [16,17]. In addition to transcription factors, it is expected that signaling pathways also contribute to temporally guided mechanisms of fate specification. For instance, neurospheres generated from cortical progenitors undergo temporal transitions that do not occur when cell–cell contact between progenitors is prevented [18]. In a different lineage, the signaling molecule transforming growth factor β2 (TGFβ2) operates as a temporal switch that promotes a late-born identity at the expense of an earlier one [19].

Another mechanism that has been proposed to influence neuronal fates is asymmetric division, which allows for the production of two different fates in sister cells derived from a common progenitor and largely involves asymmetric segregation of fate determinants during division. Asymmetric segregation of Notch pathway components has been extensively described in *Drosophila* neuronal lineages [3,20]. In vertebrates, however, asymmetric segregation of Notch interactors has been implicated in the decision to remain a progenitor or become a neuron [21,22] but not between adopting distinct neuronal fates. The decision to become a v2a or a v2b interneuron is by far the best-described vertebrate case of a Notch-dependent binary fate decision, but whether asymmetric segregation of Notch interactors plays a role in this specific instance is unclear. Indeed, although the v2a and v2b fates are produced from a common progenitor cell, the division producing these two neurons does not seem to occur at a specific angle, suggesting that a process of asymmetric segregation of fate determinants is unlikely [23]. Finally, the question of how asymmetric division is integrated with the activity of signaling pathways other than Notch and the evolution of competence in progenitors over time has yet to be thoroughly addressed.

The zebrafish pineal is a neuroendocrine organ containing two main populations of neurons: PhRs and PNs. We have previously shown that BMP and Notch cooperate during the acquisition of a generic PhR identity [6,24]. Here, we explore the mechanism underlying the specification of different PhR subtypes in the pineal gland. Using the expression of different opsin genes, we have identified three distinct subpopulations of pineal PhR. Two PhR subpopulations, which express *exorhodopsin* (*exorh*) or the *parietopsin* (*PT*), are specified sequentially, with *exorh*+ cells appearing earlier than *PT*+ cells. Reduction of Notch activity accelerates PhR production and concomitantly shifts the fate of the PhR produced from late *PT*+ to early *exorh+* identity. Gain of BMP activity, on the other hand, promotes ectopic PhR whose subtype identity depends on the time when the activation of BMP is triggered. Using time-lapse confocal microscopy, we show that PhR-generating progenitors predominantly produce sister cells with a different timing of BMP activation. In contrast, in a context in which Notch activity is reduced, BMP activation occurs either more frequently before the final division or with more symmetric timing in sister cells. Our results suggest a model in which division of a PhR-restricted progenitor generates two sister cells that activate the BMP signaling pathway at different times, resulting in the acquisition of either an “early” or “late” PhR subtype identity.

## Results

### The pineal gland contains distinct PhR subtypes that are produced in a temporal sequence

**Fig 1:**
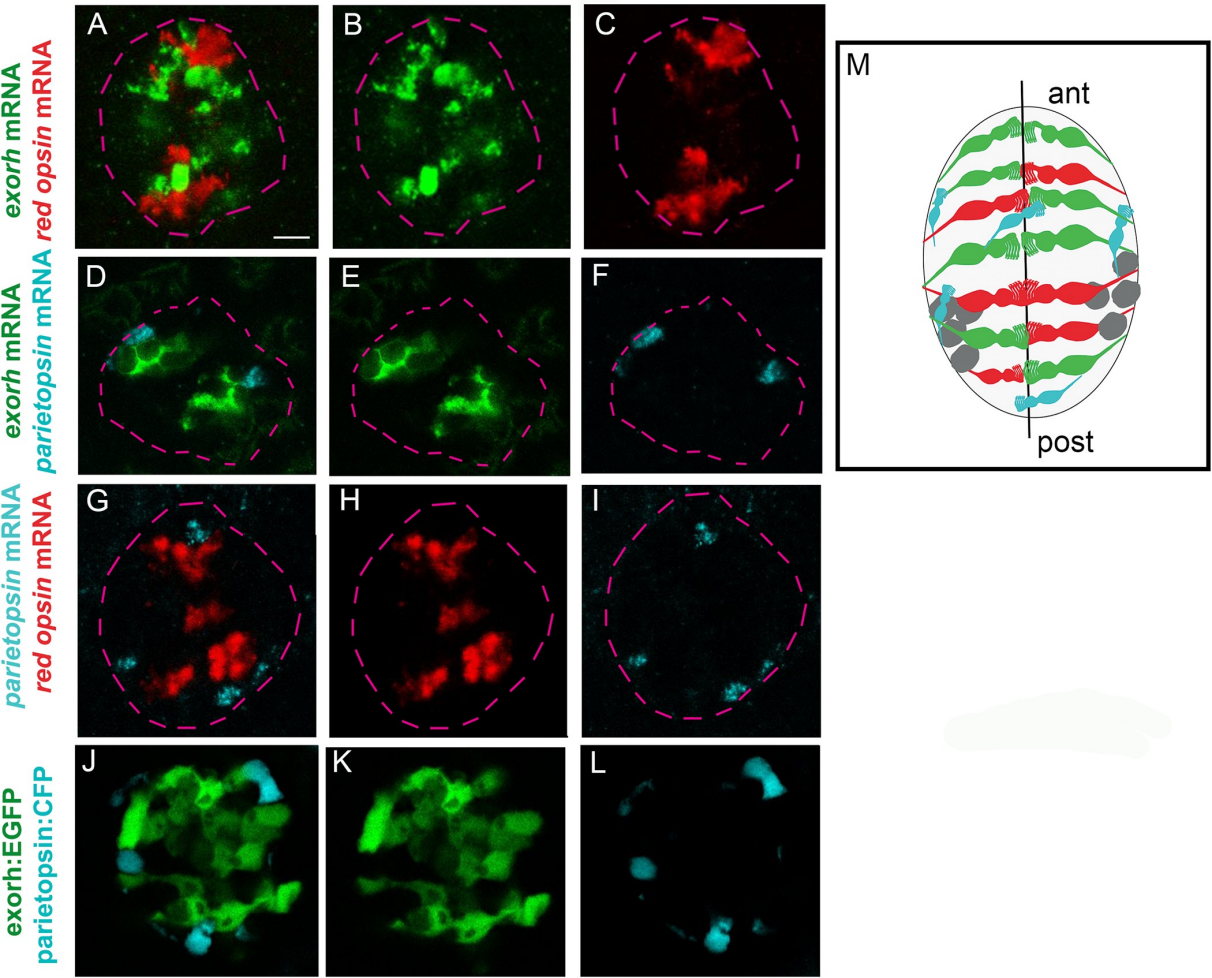
The zebrafish pineal gland contains three PhR subtypes. (A-I) Confocal sections of embryos double labeled for two of the three different opsin probes (*exorh*, *PT*, and *red opsin*). Nuclear staining (Topro3) was used to draw a mask delineating the pineal gland; these masks are shown on the panels (dotted pink lines). Embryos shown are 48 hpf. The images are falsely colored to show the *exorh+* cells in green, the *PT*+ in blue, and the *red*+ in red. Anterior (“ant”) is on the top. Scale bar represents 10 μm. (J-L) Confocal sections of live *Tg(exorh*:*EGFP)*^*ja1*^;*Tg(-2*.*2parietopsin*:*CFP)* double-transgenic embryos (exorh:EGFP; parietopsin:CFP). Embryos shown are 96 hpf. (M) Schematic representation of the organization of the pineal gland with the orientation as in A-L. The midline is shown with a black line, projection neurons are in gray, and the color code for the PhRs is as in panels A-L. Underlying data can be found in S1 Data. *exorh*, *exorhodopsin*; hpf, hours post fertilization; post, posterior; PhR, photoreceptor; *PT*, *parietopsin*.

Opsins are G-protein-coupled receptors that enable cells to sense a specific spectrum of wavelengths and intensities of light [25]. The expression of several opsins has been reported in the zebrafish pineal gland. For instance, the expression of *exorh* and *red cone opsin* (*red*) has been described in the developing and adult pineal gland using in situ hybridization [24,26], and the expression of *PT*, a green-sensitive photopigment that belongs to the so-called non-visual opsins, has been described using reverse transcription PCR (RT-PCR) [27,28]. To address whether these opsins are expressed in overlapping or restricted PhR populations, we mapped their expression relative to each other using double in situ hybridization. We found that their expression is largely restricted to distinct subpopulations of cells (Fig 1A, 1B, 1C, 1D, 1E, 1F, 1G, 1H and 1I), with only a few embryos showing one or two cells coexpressing *exorh* and *PT* or *exorh* and *red* (see quantification in Table 1); no coexpression was observed between *PT* and *red*. In parallel, we performed in situ hybridization against the endogenous opsins coupled with immunostaining for green fluorescent protein (GFP)/cyan fluorescent protein (CFP) in a previously described *Tg(exorh*:*EGFP)*^*ja1*^ transgene [29] or a transgenic reporter for the *PT* gene, *Tg(-2*.*2parietopsin*:*CFP)*, that we established for this study. In the *Tg(exorh*:*EGFP)*^*ja1*^ background, 92.3% of the GFP+ cells express the endogenous *exorh* gene (S1A, S1B and S1C Fig); in addition, extensive overlap was also observed between CFP and the endogenous *PT* gene (S1P, S1Q and S1R Fig) in *Tg(-2*.*2parietopsin*:*CFP)* transgenic embryos, suggesting that the two transgenes recapitulate endogenous expression. Finally, no overlap was detected between the two transgenes (Fig 1J, 1K and 1L, Table 1). Cross comparisons between the expression of the endogenous opsins and transgenes revealed similar levels of coexpression of exorh:EGFP with *PT* or *red* (S1D, S1E, S1F, S1G, S1H and S1I Fig) and PT:CFP with *exorh* or *red* (S1J, S1K, S1L, S1M, S1N and S1O Fig) as between the endogenous genes (Table 1). Since cells coexpressing *exorh* and *PT* or *exorh* and *red* are rare (<2 cells) and not found in all embryos, our interpretation is that these cells represent inappropriate specification events rather than hybrid cell fates.

**Table 1:** Quantification of cells coexpressing two different opsins. The frequencies of embryos showing colabeled cells and number of colabeled cells per embryo are given for each of the combinations indicated on the left. Since colabeled cells are observed in only a fraction of embryos and only a few cells in these embryos, our interpretation is that these cells do not constitute a population of PhR with mixed identity but rather represent an inappropriate specification event. Underlying data can be found in S1 Data.

	Frequency of embryos showing colabeled cells	Number of colabeled cells per embryo (range)
*exorh* mRNA/*red* mRNA	1/19	0–1
*Tg(exorh*:*EGFP)*^*ja1*^/ *red* mRNA	2/16	0–2
*exorh* mRNA/*PT* mRNA	1/16	0–1
*exorh* mRNA/ *Tg(-2*.*2parietopsin*:*CFP)*	1/5	0–2
*red* mRNA/*PT* mRNA	0/28	0
*red* mRNA*/* *Tg(-2*.*2parietopsin*:*CFP)*	0/4	0
*Tg(exorh*:*EGFP)*^*ja1*^/ *Tg(-2*.*2parietopsin*:*CFP)*	0/9	0

Abbreviations: *exorh*, *exorhodopsin*; PhR, photoreceptor; *PT*, *parietopsin*; *red*, *red cone opsin*.

Two PhR subtypes have already been described in the embryonic pineal gland, as defined by the expression of *rhodopsin* (*rhod*) and Arrestin 3a (Arr3a) [30]; these populations were called rod and cone PhRs in reference to the morphology of cells expressing these genes in the retina. We found that *rhod* mRNA expression is largely restricted to exorh:EGFP+ cells in the *Tg(exorh*:*EGFP)*^*ja1*^ transgene (S2A, S2B and S2C Fig). Similarly, we used a *Tg2PAC(opn1lw1*:*GFP*,*cxxc1*:*RFP)* transgenic line [31], in which the *red*+ population of the pineal gland is labeled with red fluorescent protein (RFP), to establish that the *red*+ fate we describe corresponds to the Arr3a+ population described previously (S2D, S2E and S2F Fig; [30]). Given that the combined number of *exorh*, *PT*, and *red+* cells accounts for the total number of PhR and that at 48–54 hours post fertilization (hpf) the average pineal gland contains 20 *exo*+ cells, 15 *red*+ cells, and 6 *PT*+ cells, we conclude that there are three subpopulations of PhR in the pineal gland, which at these stages corresponds to a composition of 48% *exorh*+, 36.6% *red*+, and 14.6% *PT*+ cells.

**Fig 2:**
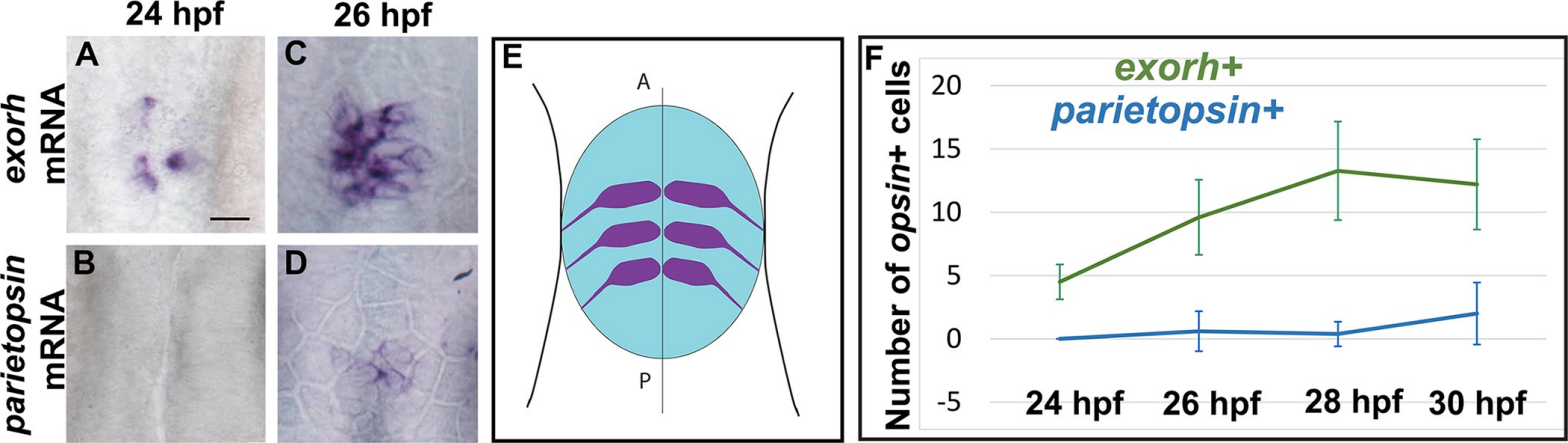
Expression of *exorh* and *parietopsin* follows a temporal sequence. (A-D) In situ hybridization for *exorh* or *parietopsin* at 24 and 26 hpf. At 26 hpf, only 2 out of 10 embryos showed *parietopsin*+ cells; in these embryos, *parietopsin* expression is restricted to the center of the pineal territory. (E) Schematic representation showing the pineal territory in blue and nascent neurons in purple, indicating the orientation of the images in (A-D); dorsal view with the anterior to the top. (F) Counts of *exorh*+ and *parietopsin*+ cells in embryos of various stages, which are indicated on the x-axis. Error bars represent SD. Number of embryos analyzed are *n* = 6, 5, 11, and 5 for *exorh*+ cells at 24, 26, 28, and 30 hpf, respectively, and *n* = 10, 10, 21, and 5 for *parietopsin*+ cells at the same stages. Scale bar represents 15 μm. Underlying data can be found in S1 Data. *exorh*, *exorhodopsin*; hpf, hours post fertilization.

As a previous study has provided insights into the specification of the *red+/arr3a+* PhR fate [30], we chose to look more closely at the *exorh+* and *PT+* populations. These subpopulations do not appear to occupy a specific region of the pineal gland except that the *PT+* population seems to be more peripheral (Fig 1D, 1E, 1F, 1G, 1H, 1I, 1J, 1K and 1L); PT+ cells are found in the center of the pineal gland at early stages (Fig 2D), suggesting that this peripheral pattern is reached secondarily. We noted, however, that whereas *exorh* expression can already be detected in the pineal at 24 hpf (Fig 2A), *PT* expression was not detected at 24 hpf and was detected only in 2 out of 10 embryos at 26 hpf (Fig 2B and 2D). Further quantification between 24 and 30 hpf confirmed that the *exorh+* and *PT+* pineal PhR populations are specified in a temporal sequence (Fig 2F).

### Accelerating PhR production favors early PhR fate at the expense of late PhR fate

**Fig 3:**
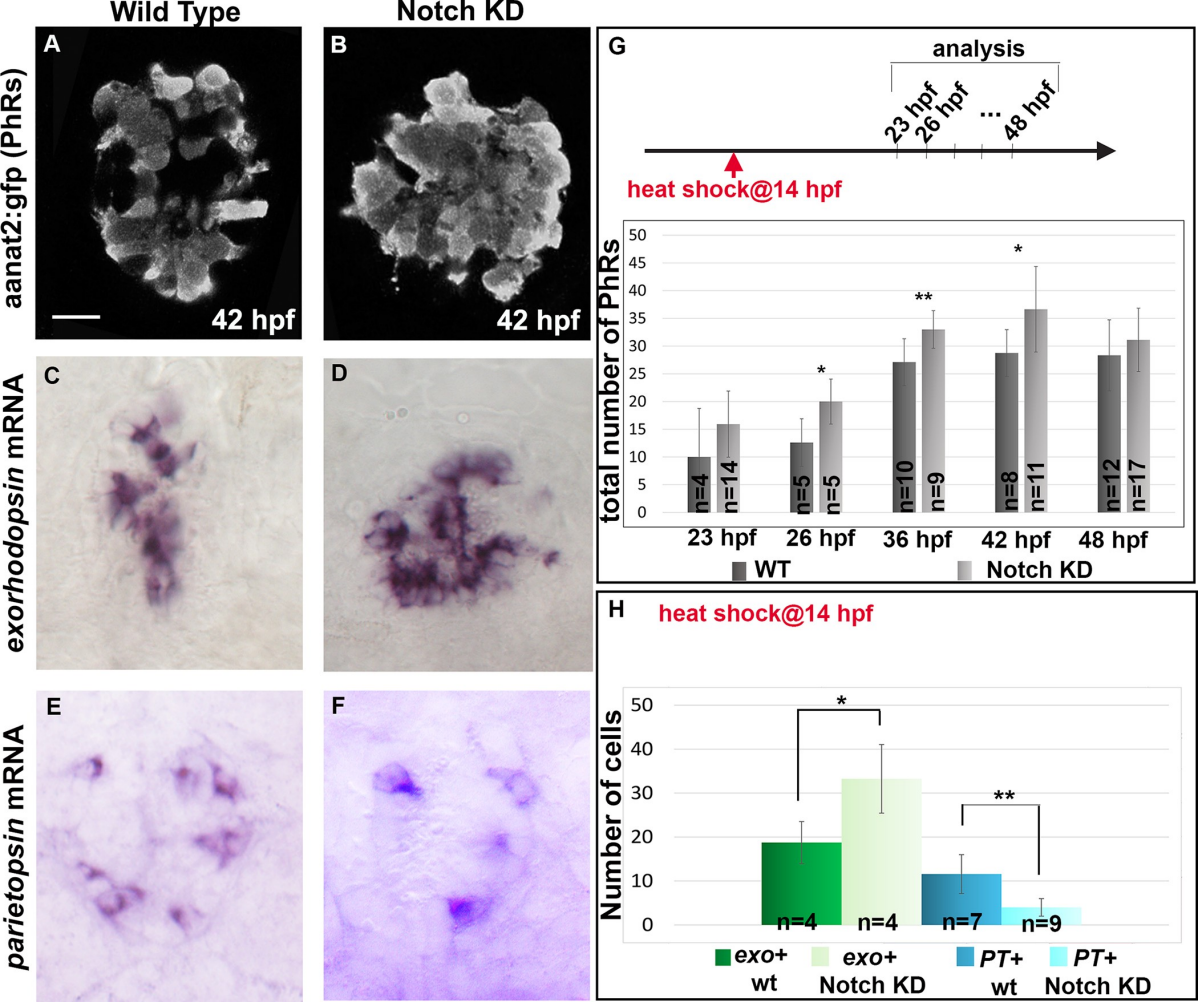
Impairing Notch activity modifies the timing of PhR differentiation and favors early PhR fate at the expense of late PhR fate. (A-B) Representative confocal sections showing the expression of the *Tg(aanat2*:*gfp)*^*y8*^ (aanat2:gfp) transgene in *Tg(hsp70l*:*dnXla*.*Rbpj-MYC)*^*vu21*^ (referred to as Notch KD) and wt siblings at 42 hpf. (C-F) Expression of *exorh* and *PT* in *Tg(hsp70l*:*dnXla*.*Rbpj-MYC)*^*vu21*^ and siblings at 48 hpf (C-D) and 66 hpf (E-F). (G) Counts of *Tg*(*aanat2*:*gfp*)^*y8*^+ cells at various stages in *Tg(hsp70l*:*dnXla*.*Rbpj-MYC)*^*vu21*^ and siblings. The stage is indicated on the x-axis. (H) Counts of *exorh+* and *PT*+ cells in *Tg(hsp70l*:*dnXla*.*Rbpj-MYC)*^*vu21*^ and sibling embryos after in situ hybridization. Stages are 48 hpf for *exorh+* and 66 hpf for *PT*+ cells. Scale bars represents 10 μm. Heat shock was performed at 14 hpf. Error bars represent SD. *p < 0.05, **p < 0.001 using a Mann Whitney test. Underlying data can be found in S1 Data. *exorh*, *exorhodopsin*; hpf, hours post fertilization; KD, knock-down; PhR, photoreceptor; *PT*, *parietopsin*; wt, wild type.

Analysis of the expression dynamics of *exorh* and *PT* suggests that these two PhR subtypes are specified at different stages during development, leading us to test the hypothesis that timing might play a role in their specification. Using a transgenic line, *Tg(hsp70l*:*dnXla*.*Rbpj-MYC)*^*vu21*^, in which a dominant negative form of the Notch effector recombination signal binding protein for immunoglobulin kappa J region (Rbpj) is overexpressed upon heat shock, we found that higher numbers of pineal cells express the pan-PhR marker anaat2:GFP [24] at 26, 36, and 42 hpf (but not at 48 hpf) relative to control siblings when heat shock was performed at 14 hpf (Fig 3A, 3B and 3G), suggesting that PhR production is accelerated upon reduction of Notch activity; this relatively late heat shock elicits only a limited effect on the PN population (S3A Fig) compared to situations in which Notch signaling has been impaired from earlier stages [24]. We next addressed whether premature PhR production modifies the subtype of PhR produced and found an increase in the number of *exorh+* cells and a reciprocal decrease in the number of *PT*+ cells (Fig 3C, 3D, 3E, 3F and 3H). Similar results were obtained using N-[N-(3,5-difluorophenacetyl)-L-alanyl]-S-phenylglycine t-butyl ester (DAPT; the gamma-secretase inhibitor) (S3B and S3C Fig), a pharmacological inhibitor of Notch activity. Taken together, these data suggest that advancing PhR production favors early PhR fate at the expense of late PhR fate in the pineal gland.

### Reducing Notch activity does not modify the production of PhR from a PhR fate-restricted progenitor

**Fig 4:**
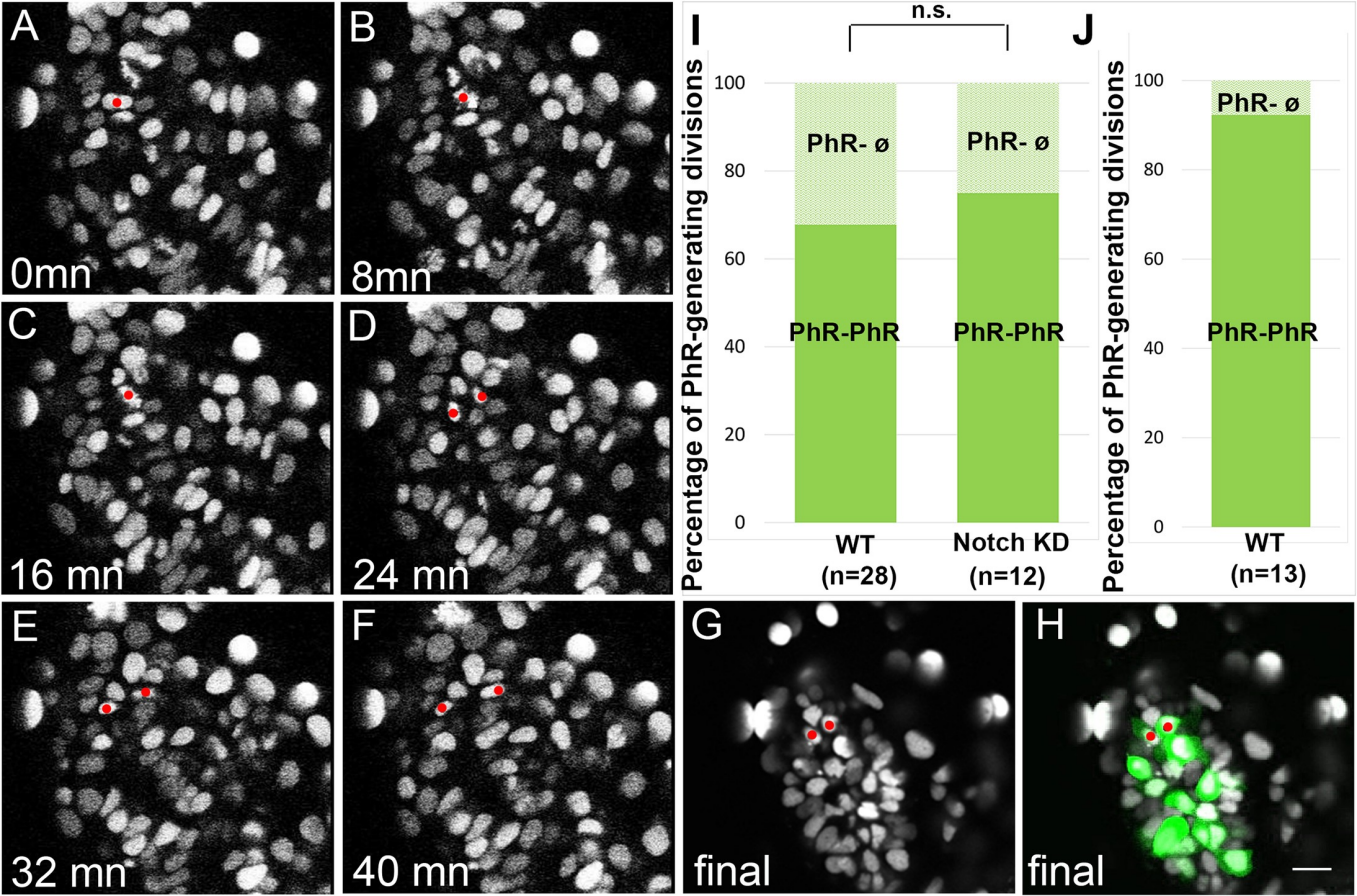
Altering Notch activity does not modify lineage relationships within the pineal. (A-H) Frames from a time-lapse dataset showing an example of a PhR–PhR division. The mother cell (A-C) and the two daughter cells (D-H) are indicated by red dots. Nuclei expressing *Histone2B*:*RFP* are shown in gray, and expression of the *Tg(aanat2*:*gfp)*^*y8*^ transgene is shown in green. Time is indicated in the lower left corner of each frame, with the first frame arbitrarily set to zero. Anterior is toward the upper right corner. Scale bar represents 10 μm. (I) Percentage of PhR-generating divisions belonging to the 2-PhR or PhR–ø cell categories in WT and *Tg(hsp70l*:*dnXla*.*Rbpj-MYC)*^*vu21*^ (Notch KD) embryos. No difference was observed between the *Tg(hsp70l*:*dnXla*.*Rbpj-MYC)*^*vu21*^ and WT embryos using a chi-squared test (n.s.). *n* indicates the number of successfully tracked cells. Thirteen and 4 embryos were used for the tracking of cells in the WT and Notch-impaired contexts, respectively. In this set of experiments, the embryos reach a final stage of 33–35 hpf. (J) Percentage of PhR-generating divisions belonging to the 2-PhR or PhR–ø cell categories in WT embryos after long acquisitions. In this set of experiments, the embryos reach a final stage of 46 hpf. *n* indicates the number of successfully tracked cells from 7 embryos. Scale bar represents 10 μm. Underlying data can be found in S1 Data. hpf, hours post fertilization; KD, knock-down; n.s., not significant; PhR, photoreceptor; WT, wild type.

To begin to address how a reduction of Notch activity could promote early production of PhR and the concomitant shift to early PhR fates, we analyzed the lineage relationships between PhR and PN in wild-type and Notch-impaired contexts. For this, we performed time-lapse confocal analyses on *Tg(aanat2*:*GFP)*^*y8*^ embryos from 15 hpf. Embryos were injected with synthetic mRNA encoding a Histone2B:RFP fusion protein at the one-cell stage to label all nuclei and permit backtracking aanat2:GFP+ cells identified at the end of the time-lapse acquisitions, and embryos were labeled for HuC/D expression at the end of the time-lapse series to identify cells that had adopted a PN fate. In an initial set of experiments, embryos were imaged for a duration of 20 hours, and based on the number of anaat2:GFP+ and HuC/D+ cells at the end of the movie, we estimate that the embryos reached 33–35 hpf at the end of the acquisition. Our results show that PhRs were born either from divisions generating 2 PhRs or a PhR and a cell with no defined identity (which we will refer to as "ø cell") (Fig 4; S1 Movie, S4 Fig, S2 Movie); ø cells do not express the PN marker HuC/D and do not divide a second time during the time-lapse series (S4 Fig, S2 Movie). Similarly, we observed that PNs were born from divisions resulting in 2 PNs or a PN and an ø cell (S5 Fig). Given that PhR–PN divisions were never detected, we hypothesized that most pineal neurons originate from fate-restricted progenitors at their last division and that in the cases of the PhR–ø divisions, the ø cell represents a future PhR that has not yet differentiated. To confirm this, we imaged embryos until they reach a stage close to 46 hpf based on the number of anaat2:GFP+ and HuC/D+ cells. Here, we found an increase from 67.8% to 92.3% of PhR–PhR divisions compared to the shorter acquisitions, supporting the notion that ø sisters of PhRs that have not yet acquired their PhR identity at 33–35 hpf will ultimately become PhRs (Fig 4I and 4J); again, no PhR–PN divisions were detected. Our results thus suggest that the vast majority of PhRs are born from fate-restricted progenitors at their last division.

To address whether impairing Notch signaling affects the lineage relationships described in wild-type embryos, we next performed similar lineage experiments in *Tg(hsp70l*:*dnXla*.*Rbpj-MYC)*^*vu21*^ embryos heat shocked at 14 hpf. We found that lineage outcomes were comparable to those in wild-type siblings, with PhR being generated from a PhR fate-restricted progenitor; the percentage of divisions generating two aanat2:GFP+ PhR was 75% in Notch-impaired embryos compared to 67.8% in the wild-type siblings (Fig 4I). We conclude that reducing Notch activity from 14 hpf onwards does not modify the lineage relationships between PhR and PN.

### Reduction of Notch activity accelerates activation of the BMP pathway

**Fig 5:**
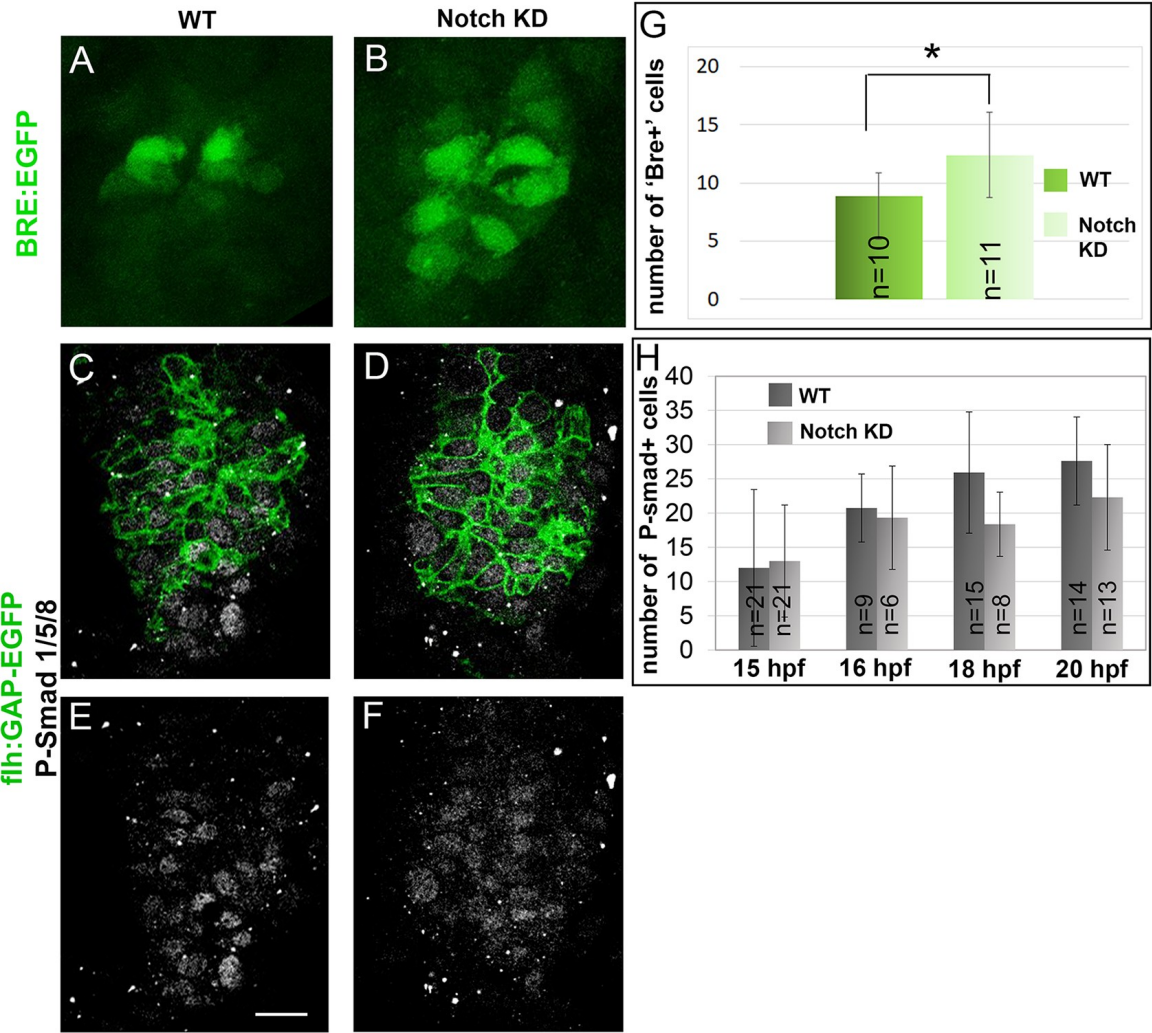
Altering Notch activity increases BMP pathway activation. (A-B) Confocal projections of WT and *Tg(hsp70l*:*dnXla*.*Rbpj-MYC)*^*vu21*^ (Notch KD) embryos at 22 hpf. Embryos are shown in dorsal views, and the *Tg(BMPRE-AAV*.*Mlp*:*EGFP)*^*mw29*^ transgene (“BRE:EGFP”) is shown in green. (C-F) Confocal sections of WT and *Tg(hsp70l*:*dnXla*.*Rbpj-MYC)*^*vu21*^ embryos at 18 hpf. The embryos are shown in dorsal views, and the *Tg(-1*.*6flh*:*GAP-EGFP)*^*u711*^ transgene (“flh:GAP-EGFP”) is shown in green, highlighting the pineal territory. Immunostaining for P-Smad1/5/8 is shown in gray. Scale bar represents 15 μm. (G) Counts of *Tg(BMPRE-AAV*.*Mlp*:*EGFP)*^*mw29*^ + (BRE+) cells in WT and *Tg(hsp70l*:*dnXla*.*Rbpj-MYC)*^*vu21*^ embryos at various stages (indicated on the x-axis). (H) Counts of P-Smad1/5/8+ cells in the *Tg(-1*.*6flh*:*GAP-EGFP)*^*u711*^+ domain at various stages, as indicated on the x-axis. Heat shock was performed at 14 hpf. Error bars represent SD. *p < 0.05 using a *t* test (G) or a Mann Whitney test (H). Underlying data can be found in S1 Data. BMP, bone morphogenetic protein; hpf, hours post fertilization; KD, knock-down; P-Smad1/5/8, phosphorylated Smad1/5/8; WT, wild type.

Activation of the BMP pathway is both necessary and sufficient to promote a PhR fate in the pineal [6]. A mechanism to explain how reducing Notch activity affects the timing of PhR determination could, therefore, involve the premature activation of the BMP pathway. To test this, we took advantage of the *Tg(BMPRE-AAV*.*Mlp*:*EGFP)*^*mw29*^ transgenic line, in which a Bmp-responsive element (BRE) from the *id1* locus has been placed upstream of GFP to create a transgene that behaves as a faithful reporter of BMP pathway activation, including in the pineal gland [32]. We counted the number of BMPRE-AAV.Mlp:EGFP+ (Bre+) cells in wild-type or *Tg(hsp70l*:*dnXla*.*Rbpj-MYC)*^*vu21*^ embryos at 22 hpf after heat shock at 14 hpf. Under these conditions, we observed an increase in BMPRE-AAV.Mlp:EGFP+ cells when the Notch pathway was inhibited (Fig 5A, 5B and 5G). A similar result was obtained using a second independent transgenic line *Tg(BMPRE-AAV*.*Mlp*:*d2EGFP)*^*mw30*^ (S6 Fig; [32]).

Activation of the BMP pathway results in the phosphorylation of Smad1/5/8, which can be detected in the pineal anlagen from around 15 hpf and peaks at 18–20 hpf [6]. We tested the possibility that Notch might act to prevent premature phosphorylation of Smad in PhR progenitors. For this, we quantified the number of phosphorylated Smad1/5/8+ (P-Smad1/5/8+) cells in the pineal of wild-type and *Tg(hsp70l*:*dnXla*.*Rbpj-MYC)*^*vu21*^ embryos using the *Tg(-1*.*6flh*:*GAP-EGFP)*^*u711*^ transgene to delineate the pineal domain. We did not observe premature Smad phosphorylation in PhR progenitors in *Tg(hsp70l*:*dnXla*.*Rbpj-MYC)*^*vu21*^ embryos heat shocked at 14 hpf (Fig 5C, 5D, 5E, 5F and 5H). Altogether, these results suggest that Notch activity affects BMP signaling in the pineal downstream of Smad1/5/8 phosphorylation and upstream of the BMP reporter *Tg(BMPRE-AAV*.*Mlp*:*EGFP)*^*mw29*^.

**Fig 6:**
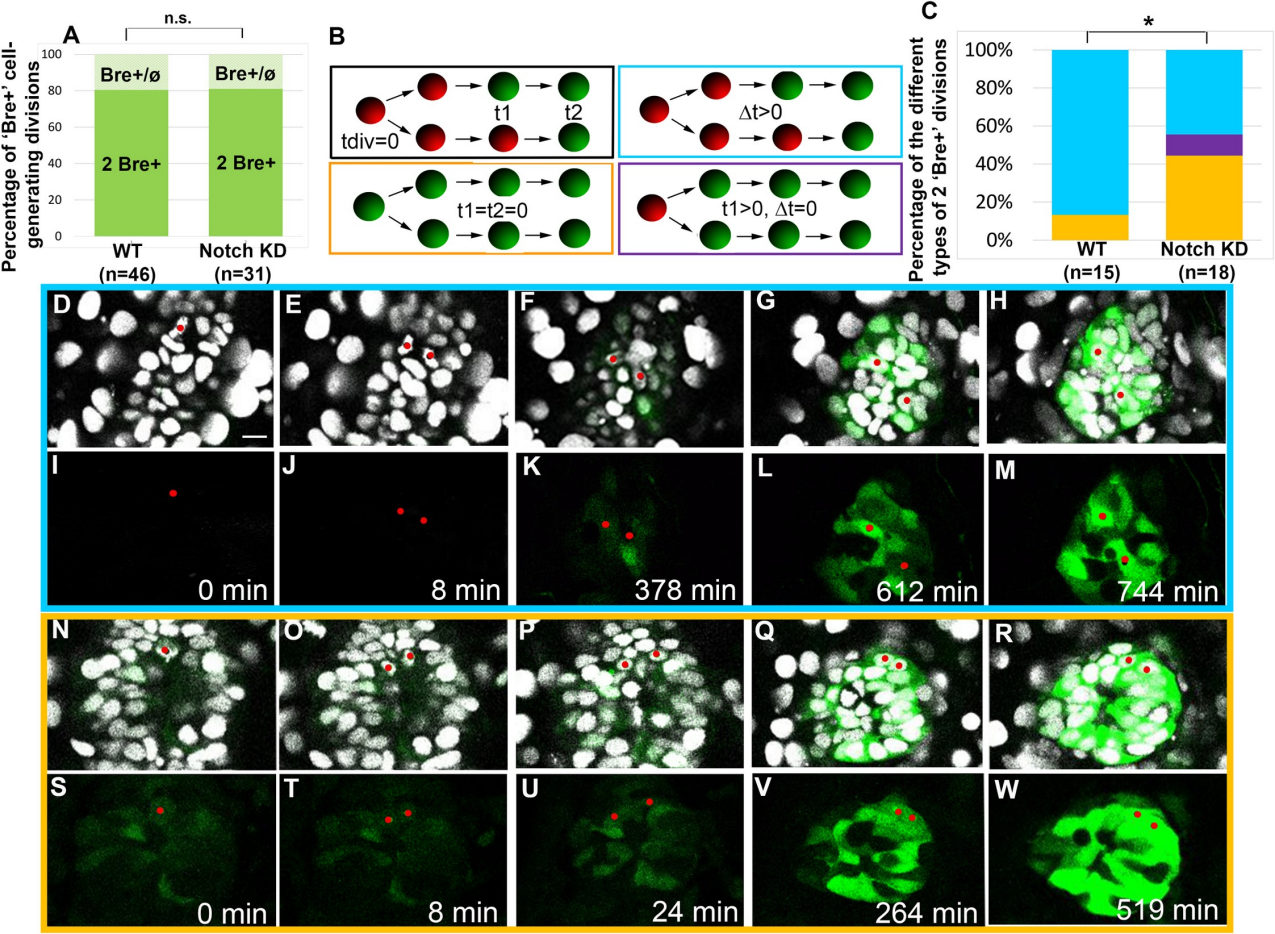
Altering Notch activity affects the heterochrony of BMP response between sister cells. (A) Percentage of cell division generating two *Tg(BMPRE-AAV*.*Mlp*:*EGFP)*^*mw29*^ + cells (2 Bre+) or a single *Tg(BMPRE-AAV*.*Mlp*:*EGFP)*^*mw29*^ + cell and an ø cell (Bre+/ø) in WT and *Tg(hsp70l*:*dnXla*.*Rbpj-MYC)*^*vu21*^ (Notch KD) embryos. No statistical difference was observed between the *Tg(hsp70l*:*dnXla*.*Rbpj-MYC)*^*vu21*^ and the WT embryos using a chi-squared test (*p* = 0.70). *n* indicates the number of successfully tracked cells from 12 WT and 8 Notch-impaired embryos. (B) Scheme explaining the timing variables extracted from time-lapse datasets as well as the color code used for the different types of 2 Bre+ divisions: blue for Δt > 0, orange for t1 = t2 = Δt = 0, and purple for t1 = t2 ≠ 0. The red circles indicate nuclei from Bre- cells, whereas green circles represent Bre+ cell nuclei. (C) Percentage of 2 Bre+ divisions belonging to the symmetric and asymmetric categories. The difference between the WT and *Tg(hsp70l*:*dnXla*.*Rbpj-MYC)*^*vu21*^ backgrounds was determined to be significant using a chi-squared test (*p* = 0.0374). *n* indicates the number of successfully tracked cells from 4 WT and 6 Notch-impaired embryos. (D-M) Frames from a time-lapse dataset obtained in a WT embryo showing an example of an asymmetric 2 Bre+ division. The frames shown in F and G correspond to t1 and t2, respectively. (N-W) Frames from a time-lapse dataset obtained in a *Tg(hsp70l*:*dnXla*.*Rbpj-MYC)*^*vu21*^ embryo showing an example of a symmetric 2 Bre+ division. The *Tg(BMPRE-AAV*.*Mlp*:*EGFP)*^*mw29*^ transgene is expressed in the progenitor prior to division. Scale bar represents 15 μm. The time of division was set as a zero. Anterior is to the top. *n* indicates the number of successfully tracked cells. Nuclei expressing Histone2B:RFP are shown in gray, and the *Tg(BMPRE-AAV*.*Mlp*:*EGFP)*^*mw29*^ transgene is shown in green. Underlying data can be found in S1 Data. BMP, bone morphogenetic protein; KD, knock-down; n.s., not significant; WT, wild type.

To explore the dynamic nature of the effect of impairing Notch, we performed time-lapse analysis using the *Tg(BMPRE-AAV*.*Mlp*:*EGFP)*^*mw29*^ transgene in a manner similar to that described above in the *Tg(aanat2*:*GFP)*^*y8*^ background. As before, we observed two types of divisions (Fig 6A): those generating two BMPRE-AAV.Mlp:EGFP+ cells (2 Bre+: 80.4%; *n* = 46 divisions) and those generating one BMPRE-AAV.Mlp:EGFP+ cell and one BMPRE-AAV.Mlp:EGFP- cell at the end of the acquisitions (Bre+/ø; 19.6%; *n* = 46 divisions). In contrast to the situation found for *Tg(aanat2*:*GFP)*^*y8*^, however, expression of the *Tg(BMPRE-AAV*.*Mlp*:*EGFP)*^*mw29*^ transgene was observed either before or after the final division. To analyze the dynamics of BMP pathway activation in pineal PhR lineages, we compared a set of temporal variables for divisions generating two BMPRE-AAV.Mlp:EGFP+ cells between wild-type and Notch-impaired conditions (Fig 6B). To generate the variables for comparison, we first set the time of final division as zero. Next, we defined the time at which the first daughter cell expresses the *Tg(BMPRE-AAV*.*Mlp*:*EGFP)*^*mw29*^ transgene as t1 and the time of activation in the second daughter cell as t2. Finally, the asynchrony in BMP pathway activation between the two daughter cells was calculated (Δt = t2 − t1). In a wild-type context, we observed two different types of divisions generating a pair of BMPRE-AAV.Mlp:EGFP+ cells: those in which the expression of the transgene occurs before division (t1 = t2 = 0) and asymmetric divisions (Δt > 0), with the latter being far more numerous (Fig 6C, 6D, 6E, 6F, 6G, 6H, 6I, 6J, 6K, 6L and 6M; S3 Movie). In *Tg(hsp70l*:*dnXla*.*Rbpj-MYC)*^*vu21*^ embryos heat shocked at 14 hpf, the final outcome of divisions was globally similar to that observed in a wild-type context, with 81% of divisions generating two BMPRE-AAV.Mlp:EGFP+ cells and 19% divisions generating one BMPRE-AAV.Mlp:EGFP+ and one BMPRE-AAV.Mlp:EGFP- cell at the end of the acquisition (*n* = 31 divisions, Fig 6A). Unlike the wild-type situation, however, impairing Notch activity led to more symmetric divisions. In particular, we observed divisions in which the expression of the transgene occurs synchronously after division (Δt = 0, t1 > 0), a case never observed in wild-type controls. Furthermore, more cases of expression of the *Tg(BMPRE-AAV*.*Mlp*:*EGFP)*^*mw29*^ transgene before division were detected in Notch-impaired than in wild-type embryos (Fig 6C, 6N, 6O, 6P, 6Q, 6R, 6S, 6T, 6U, 6V and 6W; S4 Movie).

These results suggest that pineal progenitors respond earlier to BMP signaling when Notch signaling is compromised, and the effect of Notch in this context appears to occur downstream of Smad phosphorylation.

### Timing of BMP activation controls PhR subtype identity

**Fig 7:**
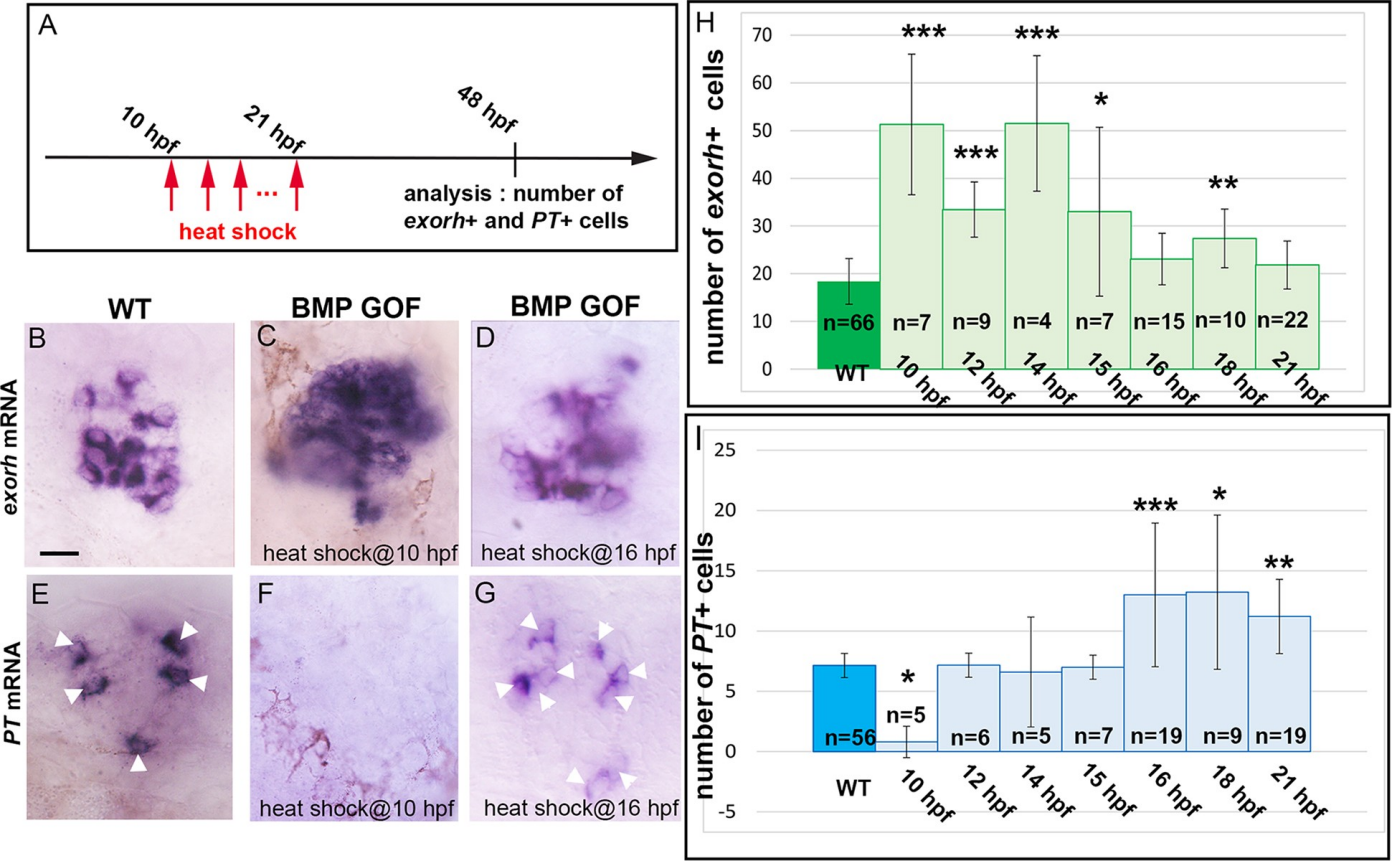
Activation of BMP signaling at different times affects PhR subtype identity. (A) Scheme of the experimental design showing the timing of the heat shocks and analysis of the number of *exorh+* and *PT*+ cells. (B-G) In situ hybridization for *exorh* and *PT* in 48-hpf WT and *Tg(hsp70*:*bmp2b)*^*fr13*^ transgenic embryos (BMP GOF) heat shocked at 10 or 16 hpf. Embryos are viewed dorsally with anterior to the top. Scale bar represents 10 μm. (H-I) Counts of *exorh+* and *PT*+ cells in WT and *Tg(hsp70*:*bmp2b)*^*fr13*^ embryos after a heat shock performed at various stages (indicated on the x-axis). Error bars represent SD. *p < 0.05. **p < 0.001. ***p < 0.0005. ****using a Kruskal-Wallis test with Dunn’s post hoc comparisons of the transgenic versus WT populations. *Tg(hsp70*:*bmp2b)*^*fr13*^ transgenic embryos were labeled BMP GOF. Underlying data can be found in S1 Data. BMP, bone morphogenetic protein; *exorh*, *exorhodopsin*; GOF, gain of function; hpf, hours post fertilization; PhR, photoreceptor; *PT*, *parietopsin*; WT, wild type.

Reducing Notch pathway activity simultaneously accelerates the response to BMP signaling and favors early PhR subtype identity. To address a potential causal link between these two observations, we tested whether the timing of BMP activity controls the subtype of PhRs formed. Forced activation of the BMP pathway in the *Tg(hsp70*:*bmp2b)*^*fr13*^ transgenic line induces ectopic PhR production [6]. We quantified the populations of each PhR subtype obtained after induction of the *Tg(hsp70*:*bmp2b)*^*fr13*^ transgene at stages between 10 and 21 hpf (Fig 7A). We detected an increase of the *exorh+* fate when the heat shock was performed relatively early (up to 18 hpf, Fig 7B, 7C, 7D and 7H). In contrast, only upon relative late heat shock (16–21 hpf) were the numbers of *PT*+ PhR increased (Fig 7E, 7G and 7I); activating BMP signaling at 10 hpf reduced the size of the PT population (Fig 7E, 7F and 7I). Together, these results suggest that activating BMP signaling at different stages produces different fates. We propose that the inhibition of the *PT*+ fate upon early BMP activation reflects the premature determination of *exorh+* PhRs, with the consequence that the PhR progenitor pool is depleted at the time when the *PT* fate should be specified.

## Discussion

**Fig 8:**
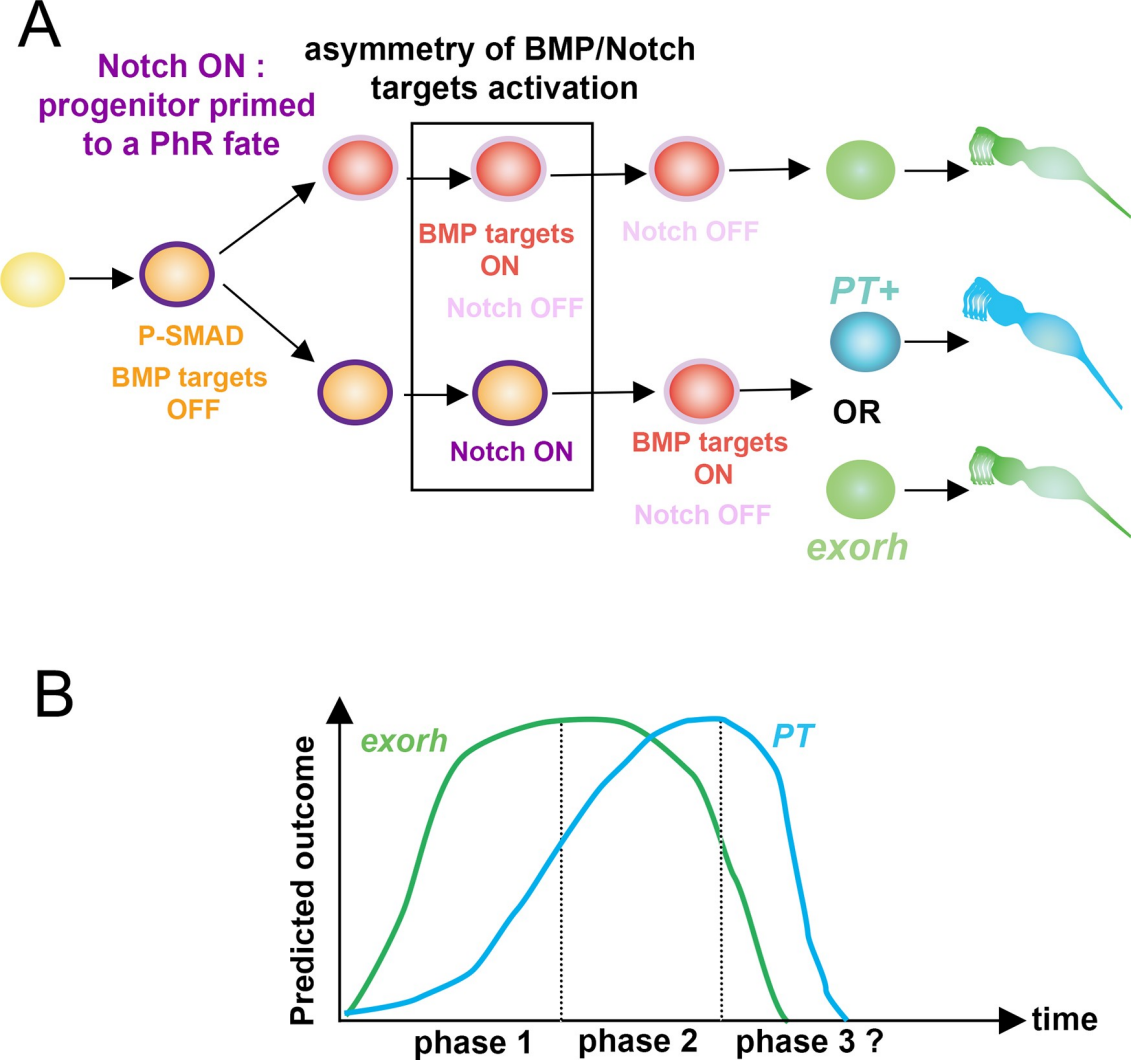
Schematized summary of the present study. (A). A naïve progenitor (yellow) activates Smad1/5/8 and Notch signaling (purple outline). This respectively primes the progenitor toward a PhR fate (pale orange: primed progenitor) and inhibits the PN fate. After division, Notch signaling introduces an asymmetry in the activation of BMP targets; one of the sister cells activates BMP targets (red cell with a pale purple outline), whereas the other does not (pale orange with a bright purple outline). Whether Notch communication occurs preferentially between sister cells remains to be determined. Notch activity is later released, resulting in the delayed activation of BMP targets compared to the sister cell. (B) Model representing the predicted outcome in terms of PhR identity relative to the timing of BMP pathway activation. Depending on the timing of the activation of BMP targets, the poised progenitors have the competence to acquire either an *exorh+* (green) or a *PT*+ fate (blue). The modeled curves represent our interpretation of the data in Fig 7G and 7H, which allowed us to distinguish three different phases. Upon early heat shock (10–15 hpf), BMP GOF mainly induces *exorh*+ cells. During the second phase (heat shock at 16–18 hpf), both *exorh*+ and *PT+* are generated. Finally, in a possible short third phase (heat shock at 21 hpf), only supernumerary *PT*+ cells are produced. BMP, bone morphogenetic protein; *exorh*, *exorhodopsin*; GOF, gain of function; hpf, hours post fertilization; PhR, photoreceptor; PN, projection neuron; P-SMAD, phosphorylated SMAD; *PT*, *parietopsin*.

In this paper, we have used specification of PhR subtype identity in the zebrafish pineal gland to address how signaling pathways underlie temporally regulated neuronal fate choice. We show that most pineal PhRs are born from a fate-restricted progenitor and that *exorh*+ PhRs are specified before ones expressing *PT*+. We show that reducing Notch pathway activity has two concomitant effects on PhR production: it accelerates PhR production and favors the early PhR fate at the expense of a later one. Finally, our results indicate that ectopic activation of BMP signaling induces PhR with an identity that depends on the timing of BMP activation. Based on these findings, we propose a model of temporal specification, which is outlined in Fig 8, and discuss its mechanistic implications.

### A temporally asymmetric division

Our analysis of BMPRE-AAV.Mlp:EGFP+ cells in a wild-type context suggests that in nearly 90% of cases, the divisions that generate these cells are asymmetric in the sense that the activation of BMP activity does not occur simultaneously in sister cells. The timing of BMP pathway activation in these divisions becomes more symmetric in a context in which Notch activity is reduced. Indeed, we observe that reduction of Notch activity increases the percentage of cells that activate BMP prior to division, suggesting that there is an inhibition of BMP activity by Notch at the level of the PhR progenitor. In this regard, the approximately 11% of divisions generating sister cells that simultaneously begin expressing GFP after mitosis in *Tg(hsp70l*:*dnXla*.*Rbpj-MYC)* embryos could represent cells in which *gfp* is transcribed already in the progenitor but GFP protein is not yet detected. We propose that an important prerequisite for temporally asymmetric divisions is the prevention of a premature fate decision within the progenitor. In invertebrates, sister cells in neuronal lineages often communicate via Notch to acquire their fates [3,20]. At this point, it is unclear whether Notch communication is occurring preferentially between sister cells in the pineal gland rather than between neighbors of unrelated lineage.

### Notch and BMP: A complex relationship

Results presented here suggest an intricate interplay between Notch and BMP activities while also expanding the roles for BMP signaling during development of the pineal gland. The pathway is first required to define the dorsoventral position of the pineal anlage [33]. In a second step, it is required for the proliferation of pineal progenitors [6]. These two BMP activities do not seem to be controlled by Notch. BMP and Notch are then required to specify a generic PhR identity and, subsequently, specific PhR subtypes. The pleiotropic effects of BMP signaling on pineal development considerably complicate the identification of BMP targets relevant for each of these processes. Indeed, although classical BMP targets such as *id1* and *msxb/c/e* are expressed in the pineal gland [32,34], it is unclear whether they underlie specific roles for BMP signaling.

Our results have led us to propose the following model (Fig 8). In a wild-type context, Smad1/5/8 is phosphorylated in fate-restricted PhR progenitors but at a level that does not translate into the activation of BMP target genes, because of inhibition exerted by Notch activity. Notch pathway activity thus restrains the progenitor from adopting a PhR fate. After division, this inhibition is progressively released, although not synchronously, and this allows for the production of *exorh+* and *PT*+ PhR, whose fates are dependent on the timing at which they activate the BMP pathway. How PhR precursors evolve from generating only *exorh+* cells early to also generating *PT*+ fates later remains an open question. Future studies concerning how different sets of BMP target genes evolve over time should shed light on this.

We have previously shown that during the PN/PhR fate decision, BMP is required for proper activation of Notch pathway targets, which in turn inhibit the PN fate in the future PhR [6]. The results presented here suggest a previously unanticipated complexity in Notch/BMP cross talk. Indeed, how can we reconcile that BMP signaling is required to activate Notch targets during the PN/PhR fate choice but that Notch pathway activation is required to prevent premature BMP activation during PhR subtype specification? One hypothesis relies on the existence of complexes containing the intracellular domain of Notch (NICD)/Rbpj and P-Smad1/5/8. Our analysis of P-Smad1/5/8 in wild-type and Notch-impaired embryos suggests that Notch inhibits the response to BMP downstream of Smad phosphorylation. The published BMP response element used in this study does not contain Rbpj binding sites [32]. This suggests that the effect of Rbpj on the *Tg(BMPRE-AAV*.*Mlp*:*EGFP)*^*mw29*^ transgene is either indirect or involves trapping of P-Smad1/5/8 species in inactive complexes. It has been shown that NICD coprecipitates with Smad1 in the presence of the coactivators P300 and the p300/CBP associated factor (P/CAF) [35]. These interactions have been proposed to reinforce the activation of Notch target genes in neural cells. Similarly, complexes containing NICD and Smad1/5/8 have been detected in cerebrovascular endothelial cells, where they are proposed to activate transcription of N-cadherin via an Rbpj binding site [36]. We propose that NICD/Rbpj/P-Smad1/5/8 complexes in the pineal participate both in the transcription of Notch target genes, as previously suggested [6], but also prevent the activation of BMP targets through a squelching mechanism. In this model, simultaneous activation of Notch and BMP receptors would lead to the formation of NICD/Rbpj/P-Smad1/5/8 complexes, but these complexes would first go to Notch targets, perhaps because of the higher affinity of Rbpj for its target sequences in the genome. The presence of Smad1/5/8 in these complexes would help transactivation of Notch targets, as previously described [35,36]. In a second step, either owing to a reduction in the level of ligands available to bind the Notch receptor or to a Notch-inhibiting signal, Notch activation would progressively decrease while activation of the BMP receptor would be maintained, and this would permit the activation of BMP target genes.

Finally, whereas, to our knowledge, our study provides the first evidence that temporal control of BMP activity is crucial for specification of postmitotic neurons, the importance of a proper timing of BMP activity for specification of progenitor pools has been previously demonstrated in the dorsal spinal cord. In this case, rather than the timing of onset of BMP activity, duration of the exposure to BMP ligands seems the most important variable [37]. It is at present unclear whether the duration of exposure to BMP activity also plays a role in the specification of pineal PhRs.

### A widespread role for Notch in controlling other signaling pathways activities

Examples of vertebrate neuronal lineages in which loss of Notch activity promotes early fates at the expense of late ones are already known [38–43]. In contrast, the mechanisms behind these effects of Notch are unclear. Notch could simply act to slow down neurogenesis and thus indirectly prevent temporal transitions. Alternatively, the pathway could play a more active role in triggering such transitions by promoting switches in the expression of temporal transcription factors in a manner analogous to what has been proposed for TGFβ2 in the hindbrain [19] or by regulating the competence of progenitors to respond to specific signaling pathways whose activity is interpreted differently over time, as is the case for pineal PhR subtype specification. Neurons in the left and right habenular nuclei of zebrafish develop with a temporal asymmetry in identity [38]. In this system, Notch has been proposed to promote identity through a general effect on the timing of neurogenesis [38]. Wnt activity was also recently shown to be necessary for the acquisition of right-sided neuronal phenotypes in the zebrafish habenulae [44]. Thus, an alternative hypothesis would be that Notch acts more directly to limit the competence to respond to Wnt activity. Along the same line, as Notch has been shown to facilitate Sonic hedgehog signaling during the specification of neural fates in the ventral spinal cord [4], it would be interesting to address whether a similar cross talk operates during the temporal neural to glial fate switch occurring within some of these ventrally specified progenitors, as such a switch has been shown to depend on a late burst of Sonic hedgehog activity [45].

### Conclusion

Numerous diseases that affect retinal PhRs and lead to blindness have been described. One promising area of research aimed at treating these conditions involves generating PhRs in vitro with the longer-term aim of developing cell replacement strategies [46]. A recent study on the induction of retina from human induced pluripotent stem cells (iPSCs) in culture suggests that inhibition of Notch activity accelerates the production of PhRs [47]. These observations led the author to envisage using pharmacological inhibitors of Notch activity to accelerate the production of these cells. A question that has not yet been answered in this system, however, is whether modifications in timing are concomitant with fate changes in the types of PhRs that are induced, as we describe in the present study. We propose that the zebrafish pineal gland provides a powerful model for understanding molecular mechanisms driving neuronal subtype specification and for addressing specific questions concerning the establishment of distinct PhR identities.

## Material and methods

### Ethics statement

All animals were handled in the CBI fish facility, which is certified by the French Ministry of Agriculture (approval number A3155510). The project was approved by the French Ministry of Teaching and Research (agreement number APAFIS#3653–2016011512005922), in accordance with the guidelines from the European directive on the protection of animals used for scientific purposes (2010/63/UE), French Decret 2013–118.

### Experimental model and subject details

Embryos were reared at 28.5°C and staged according to standard protocols [48]. The *Tg(exorh*:*EGFP)*^*ja1*^ [29], *Tg(hsp70l*:*dnXla*.*Rbpj-MYC)*^*vu21*^ [49], *Tg(aanat2*:*GFP)*^*y8*^ [50], *Tg(BMPRE-AAV*.*Mlp*:*EGFP)*^*mw29*^ and *Tg(BMPRE-AAV*.*Mlp*:*d2EGFP)*^*mw30*^ [32], *Tg(-1*.*6flh*:*GAP-EGFP)*^*u711*^ [51], and *Tg(hsp70*:*bmp2b)*^*fr13*^ [52] have been described previously.

Conditions of heat shock were as follows: *Tg(hsp70*:*bmp2b)*^*fr13*^ 30 minutes at 37°C and *Tg(hsp70l*:*dnXla*.*Rbpj-MYC)*^*vu21*^ 30 minutes at 39.5°C.

Genotyping of *Tg(hsp70l*:*dnXla*.*Rbpj-MYC)*^*vu21*^ was performed either using immunohistochemistry against the Myc epitope tag or via a nested PCR with the following couples of oligos:

5′-GCCACTTTTGTCCCTGATGC-3′

5′-CTTTTTACATGTGGACTGCC-3′

and then,

5′-CCTTCCAGGTTCAGCTGCTG-3′

5′-CGGGCATTTACTTTATGTTGC-3′.

Genotyping of *Tg(hsp70*:*bmp2b)*^*fr13*^ was performed as described in [6].

To generate an in vivo marker of *PT*-expressing PhRs in the pineal gland, we amplified a 2.2-kb fragment of *PT* regulatory sequences immediately upstream of the ATG by PCR using the following oligos:

5′-CGACCTCGAGGTAGGCCTACATTAAGCGAT-3′

5′-GCGCGGATCCGATGATTCGGAATGATCTTC-3′.

The resulting fragment was subcloned into a pBS-I-SceI backbone upstream of the coding region of CFP. To generate the *Tg(-2*.*2parietopsin*:*CFP)* transgenic line, this construct was coinjected with I-SceI meganuclease into freshly fertilized embryos following previously described protocols [53]. The presence of successfully inserted transgenes was assessed using PCR with the following oligonucleotides:

5′-GGACACGCTGAACTTGTGG-3′

5′-GGTACTTGTTCAGATGGCTG-3′.

### Method details

#### DAPT treatment

Treatment with the pharmacological agent DAPT was performed as described in [24].

#### In situ hybridization and immunohistochemistry

In situ hybridization and immunohistochemistry were performed as previously described [6,24]. Double in situ hybridization were performed using fluorescein-dUTP and DIG-dUTP probes according to a similar protocol. Embryos were incubated in a mixture of anti-dig alkaline phosphatase (AP; 1/2,500; Roche) and anti-fluorescein peroxidase (POD, 1/500; Roche) and revealed with the TSA Plus System (TSA-Fluorescein, Perkin Elmer) followed by a regular Fast Red staining (Sigma). A more detailed protocol is available upon request.

The plasmids used to generate probes for *exorh*, *rhod*, and *red* (*lws1*) were provided by D. Whitmore and S. Kawamura, respectively. To generate *PT* probes, we performed a PCR on zebrafish genomic DNA using the following oligos to amplify a fragment of the *PT* coding sequence:

5′-CCGGGATCCAGATCATTCCGAATCATC-3′

5′-CGCGCTCGAGCCCTCGTAGTTAGTGACGG-3′.

This fragment was inserted into pCS2 using BamH1 and Xho1. An antisense probe was generated using BamH1 and T7.

Immunohistochemistry was performed with the following antibodies: rabbit GFP/CFP (Torrey Pines Biolabs), rabbit Phospho-Smad1/5/8 (Cell Signaling), HuC/D (Molecular Probes), and FRet43 (Arr3a antibody, zpr1; ZIRC). Immunohistochemistry against the Myc epitope tag was performed using the 9E10 antibody (CliniSciences).

#### Time-lapse analysis

Embryos were injected with synthetic mRNA encoding Histone2B:RFP at the one-cell stage. At 14 hpf, injected embryos were mounted in 0.7% agarose, and time-lapse datasets were generated on either a Leica SP5 or Zeiss LSM710 inverted confocal microscope. For the “short acquisitions” (Fig 4A–4H; Fig 6, S1 Movie, S2 Movie, S3 Movie, S4 Movie, S4 Fig, S5 Fig), stacks of 50 or 60 μm were obtained every 8 minutes during the first 16 hours and then every 15 minutes for a period that varied depending on the experiment. For the “long acquisitions” (Fig 4I), stacks of 60 μm were obtained every 8 minutes during the first 16 hours and then every 30 minutes for a period of 24 hours. In this set of experiments, the embryos were maintained at a temperature of 30°C and imaged one last time 7 hours after the end of the acquisition to identify late-specified cells. A division generating one pineal cell and one parapineal cell was observed but discarded from the statistics because of the late differentiation timing of the parapineal compared to the pineal. At the end of the acquisitions, embryos were recovered from the agarose and fixed prior to immunohistochemistry. Cells were manually tracked using ImageJ. Since the tracking was made on a z-stack, we reconstituted movies of a single plane per time point using an ImageJ macro written specifically for this study, which is available as S3 Data.

To quantify the onset of GFP fluorescence from the *Tg(BMPRE-AAV*.*Mlp*:*EGFP)*^*mw29*^ transgene in these experiments, we used both visual inspection and quantification on a representative sample of the data. Acquisition was realized using invariant confocal variables (laser power, pinhole, gain, and offset) to avoid introducing variation. Post hoc quantification was performed using a constant ROI of 34 μm^2^ applied on a single plane in the middle of the nucleus of each cell and the ImageJ plugin ROI manager, which confirmed a clear difference between the integrated density of fluorescence in progenitors expressing the transgene prior to division and those that do not. In parallel, we reconstituted the kinetics of fluorescence onset before and after division in the progenitors and related daughter cells. Comparison between visual detection and quantitative methods suggests that fluorescence was detected when its integrated density was ≥750 arbitrary units. Such comparison also confirmed that we were able to capture the synchronous or asynchronous nature of the appearance of GFP between sister cells.

### Quantification and statistical analysis

For experiments in which numbers of cells were assessed in fixed material, we assessed statistical significance using either a *t* test, a Mann Whitney test, or a Kruskal-Wallis test with Dunn’s post hoc comparisons (Fig 7). Statistical tests and number of embryos used are stated in each figure and/or figure legend.

## Supporting information

S1 Data Excel spreadsheet containing, in separate sheets, the underlying numerical data and statistical analysis for Figs 1, 2, 3G, 3H, 4, 5E, 5, 6A, 6C, 7H and 7I. (ODS)

S2 Data Excel spreadsheet containing, in separate sheets, the underlying numerical data and statistical analysis for S1 Fig, S2 Fig, S3A Fig, S3B Fig, S3C Fig and S6 Fig. (ODS)

S3 Data Word file containing the two scripts necessary to run the ImageJ macro to reconstitute a movie of a single plane per time point from a z-stack movie. (ODT)

S1 Fig (Related to Fig 1). **Expression of the *Tg(exorh*:*EGFP)***^***ja1***^ **and *Tg(-2*.*2parietopsin*:*CFP)* transgenes recapitulates *exorh* and *PT* expression.** Confocal sections showing double-labeling for *exorh*, *PT*, or *red opsin* by in situ hybridization and either the *Tg(exorh*:*EGFP)*^*ja1*^ (exorh:EGFP in green, A-I) or the *Tg(-2*.*2parietopsin*:*CFP)* transgene (PT:CFP in cyan, J-R). (A-F) Forty-eight hpf; (G-I) 54 hpf;(J-O) stage 63 hpf; (P-R) stage 72 hpf. Anterior is in the upper right corner. Scale bar represents 10 μm. White arrowheads point at double-labeled cells. A total of 92.3% of *Tg(exorh*:*EGFP)*^*ja1*^*+* cells were *exorh+* (*n* = 4), and 78.6% *Tg(-2*.*2parietopsin*:*CFP)+* cells were *PT+* (*n* = 5). (S) Schematic representation of the organization of the pineal gland. Color code is the same as for Fig 1. Orientation is the same as for panels (A-R). Underlying data can be found in S2 Data. hpf, hours post fertilization; *exorh*, *exorhodopsin*; *PT*, *parietopsin*.
(TIF)

S2 Fig (Related to Fig 1). **Comparison between the present description of pineal fates and previously reported “rod” and “cone” PhR fates in the pineal.** (A-C) Confocal sections of 48-hpf embryos stained by in situ hybridization against *rhodopsin* (in magenta) and the *Tg(exorh*:*EGFP)*^*ja1*^ transgene (exorh:EGFP, in green). A high percentage of coexpression was observed between *rhodopsin* and the *Tg(exorh*:*EGFP)*^*ja1*^ transgene (“exorh:EGFP”; 76.5% of *rho*+/EGFP+ cells over the total number of EGFP+ cells, *n* = 11). (D-F) Confocal sections of 72-hpf embryos labeled with RFP (in red) and Arr3a (in gray) antibodies in a *Tg2PAC(opn1lw1*:*GFP*,*cxxc1*:*RFP)* transgenic line (“lws:RFP”; in red), which labels cells expressing *red cone opsin* [31]. All Arr3a+ cells were also RFP+ (from *n* = 10 embryos). White arrowheads point at double-labeled cells. Scale bar is 15 μm. Underlying data can be found in S2 Data. Arr3a, Arrestin 3a; EGFP, enhanced green fluorescent protein; *exorh*, *exorhodopsin*; hpf, hours post fertilization; RFP, red fluorescent protein.
(TIF)

S3 Fig (Related to Fig 3). **Impairing Notch activity affects the timing of PhR production and the fate of the PhRs produced.** (A) Counts of HuC/D+ PN cells in *Tg(hsp70l*:*dnXla*.*Rbpj-MYC)*^*vu21*^ (Notch KD) and WT embryos at 48 hpf. Heat shock was performed at 14 hpf. (B) Counts of *Tg*(*aanat2*:*gfp*)^*y8*^+ PhRs at various stages in DAPT versus mock-treated (DMSO) embryos. Treatment was performed at 9 hpf. The stage of analysis is indicated on the x-axis. A decrease in the number of PhRs at 48 hpf is observed upon treatment with DAPT, a phenotype we previously attributed to a greater occurrence of cell death [24]. (C) Counts of *exorh+* and *PT*+ cells in DAPT versus mock-treated (DMSO) embryos after in situ hybridization. For *exorh*, embryos were 48 hpf, and for *PT*, embryos were 54 hpf. Error bars represent SD. *p < 0.05, **p < 0.001, ***p < 0.0005 using a Mann Whitney test. Underlying data can be found in S2 Data. DAPT, N-[N-(3,5-difluorophenacetyl)-L-alanyl]-S-phenylglycine t-butyl ester; *exorh*, *exorhodopsin*; hpf, hours post fertilization; KD, knock-down; PhR, photoreceptor; *PT*, *parietopsin*; WT, wild-type.
(TIF)

S4 Fig (Related to Fig 4). **A division generating one PhR and one ø cell.** (A-G) Frames from a time-lapse dataset showing a representative PhR–ø division. The mother and daughter cells are indicated with a black dot. Histone2B:RFP-labeled nuclei are in gray, whereas *Tg(aanat2*:*gfp)*^*y8*^ is shown in green, and immunostaining against HuC/D is in cyan. Anterior is toward the upper left corner. The ø cell is not a PN, as judged by the absence of HuC/D (G). (H) Schema indicating the organization and the orientation of the pineal. Anterior is toward the upper left corner, as in the individual frames. PhRs are in green and PNs in cyan. Scale bar is 15 μm. PhR, photoreceptor; PN, projector neuron.
(TIF)

S5 Fig (Related to Fig 4). **PN are born from fate-restricted progenitors.** Frames from a time-lapse dataset showing examples of representative PN–ø (A-C) and PN–PN (D-F) divisions. In the case of the PN–ø division, the sister cells end up on different z planes, which are shown in B and C, respectively. The sister cells are indicated with a red dot. Histone2B:RFP+ nuclei are in gray, *Tg(aanat2*:*gfp)*^*y8*^ is shown in green, and immunostaining against HuC/D is in cyan. Anterior is toward the upper left corner. A total of 3 PN–PN and 6 PN–ø (*n* = 9 divisions) were successfully tracked. Scale bar is 20 μm. PhR, photoreceptor; PN, projector neuron.
(TIF)

S6 Fig (Related to Fig 5). **Alteration of Notch activity modifies the expression of the *Tg(BMPRE-AAV*.*Mlp*:*d2EGFP)***^***mw30***^ **transgene.** (A-B) Confocal projections of WT and *Tg(hsp70l*:*dnXla*.*Rbpj-MYC)*^*vu21*^ (Notch KD) embryos at 22 hpf. Embryos are shown in dorsal views. The *Tg(BMPRE-AAV*.*Mlp*:*d2EGFP)*^*mw30*^ transgene (BRE:d2EGFP) is shown in green. Scale bar is 15 μm. (C) Counts of dGFP+ cells in WT and *Tg(hsp70l*:*dnXla*.*Rbpj-MYC)*^*vu21*^ (Notch KD) embryos at 23 hpf. Underlying data can be found in S2 Data. Heat shock was performed at 14 hpf. Error bars represent SD. *p < 0.05 using a *t* test. Underlying data can be found in S2 Data. D2EGFP, destabilized enhanced green fluorescent protein; KD, knock-down; hpf, hours post fertilization; WT, wild-type.
(TIF)

S1 Movie (Related to Fig 4). **A division generating two PhRs.** A time-lapse movie showing fluorescent nuclei labeled with the Histone2B:RFP (in gray). A division generating two PhRs (PhR–PhR) is highlighted with black dots. Frames of this dataset are shown in Fig 4. PhR, photoreceptor.
(AVI)

S2 Movie (Related to Fig 4). **A division generating one PhR and one ø cell.** A time-lapse movie showing fluorescent nuclei labeled with the Histone2B:RFP (in gray). A division generating one PhR and one ø cell is highlighted with black dots. Frames of this dataset are shown in S5 Fig. PhR, photoreceptor.
(AVI)

S3 Movie (Related to Fig 6). **A division generating sister cells with an asynchronous BMP response in a wild-type embryo.** A time-lapse movie showing fluorescent nuclei labeled with Histone2B:RFP in gray and the *Tg(BMPRE-AAV*.*Mlp*:*EGFP)*^*mw29*^ transgene in green. Frames of this movie are shown in Fig 7D–7H. BMP, bone morphogenetic protein.
(AVI)

S4 Movie (Related to Fig 6). **A BMP-responsive progenitor filmed in a *Tg(hsp70l*:*dnXla*.*Rbpj-MYC)***^***vu21***^ **embryo.** A time-lapse movie showing fluorescent nuclei labeled with Histone2B:RFP in gray and the *Tg(BMPRE-AAV*.*Mlp*:*EGFP)*^*mw29*^ transgene in green. Frames of this movie are shown in Fig 7I–7M. BMP, bone morphogenetic protein.
(AVI)

## Abbreviations

Arr3a: Arrestin 3a
BMP: bone morphogenetic protein
BRE: Bmp-responsive element
Casz1: castor zinc finger 1
CFP: cyan fluorescent protein
DAPT: N-[N-(3,5-difluorophenacetyl)-L-alanyl]-S-phenylglycine t-butyl ester
exorh: *exorhodopsin*
Foxg1: forkhead box G1
GFP: green fluorescent protein
GOF: gain of function
hpf: hours post fertilization
Ikzf1: IKAROS family zinc finger 1
iPSC: induced pluripotent stem cell
KD: knock-down
NICD: intracellular domain of Notch
n.s.: not significant
P/CAF: p300/CBP associated factor
PhR: photoreceptor
PN: projection neuron
P-Smad1/5/8: phosphorylated Smad1/5/8
PT: *parietopsin*
red: *red cone opsin*
Rbpj: recombination signal binding protein for immunoglobulin kappa J region
RFP: red fluorescent protein
rhod: *rhodopsin*
RT-PCR: reverse transcription PCR
TGFβ2: transforming growth factor β2
