# Supplementary figures and images for: A Notch-mediated, temporal asymmetry in BMP pathway activation promotes photoreceptor subtype diversification
Source: PLoS Biol. 2019 Jan 31;17(1):e2006250. doi: 10.1371/journal.pbio.2006250 (PMC6372210; doi:10.1371/journal.pbio.2006250)

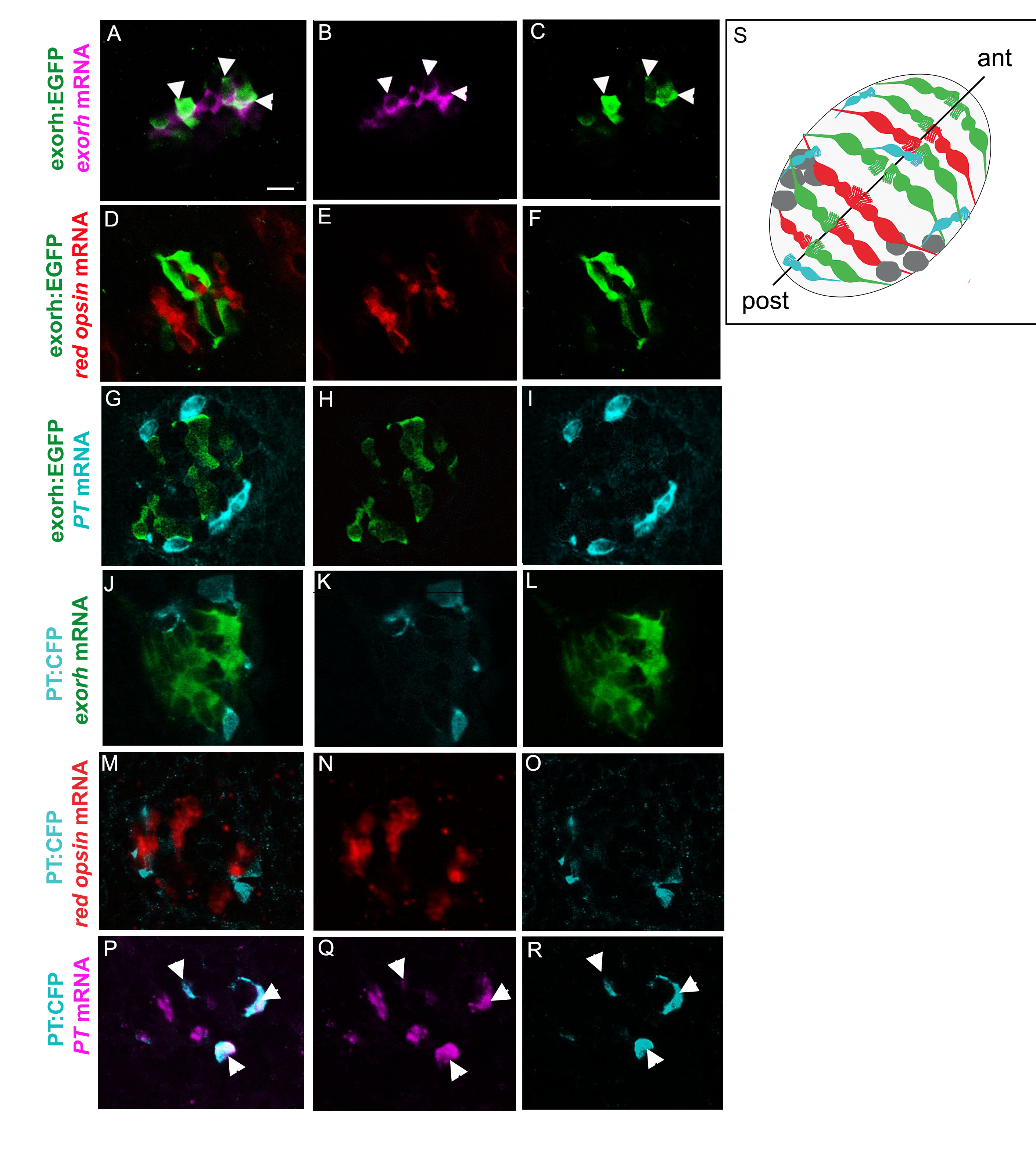

Supplement: S1 Fig — Expression of the Tg(exorh:EGFP)ja1 and Tg(-2.2parietopsin:CFP) transgenes recapitulates exorh and PT expression. Confocal sections showing double-labeling for exorh, PT, or red opsin by in situ hybridization and either the Tg(exorh:EGFP)ja1 (exorh:EGFP in green, A-I) or the Tg(-2.2parietopsin:CFP) transgene (PT:CFP in cyan, J-R). (A-F) Forty-eight hpf; (G-I) 54 hpf;(J-O) stage 63 hpf; (P-R) stage 72 hpf. Anterior is in the upper right corner. Scale bar represents 10 μm. White arrowheads point at double-labeled cells. A total of 92.3% of Tg(exorh:EGFP)ja1+ cells were exorh+ (n = 4), and 78.6% Tg(-2.2parietopsin:CFP)+ cells were PT+ (n = 5). (S) Schematic representation of the organization of the pineal gland. Color code is the same as for Fig 1. Orientation is the same as for panels (A-R). Underlying data can be found in S2 Data. hpf, hours post fertilization; exorh, exorhodopsin; PT, parietopsin. (TIF) [file pbio.2006250.s004.tif]

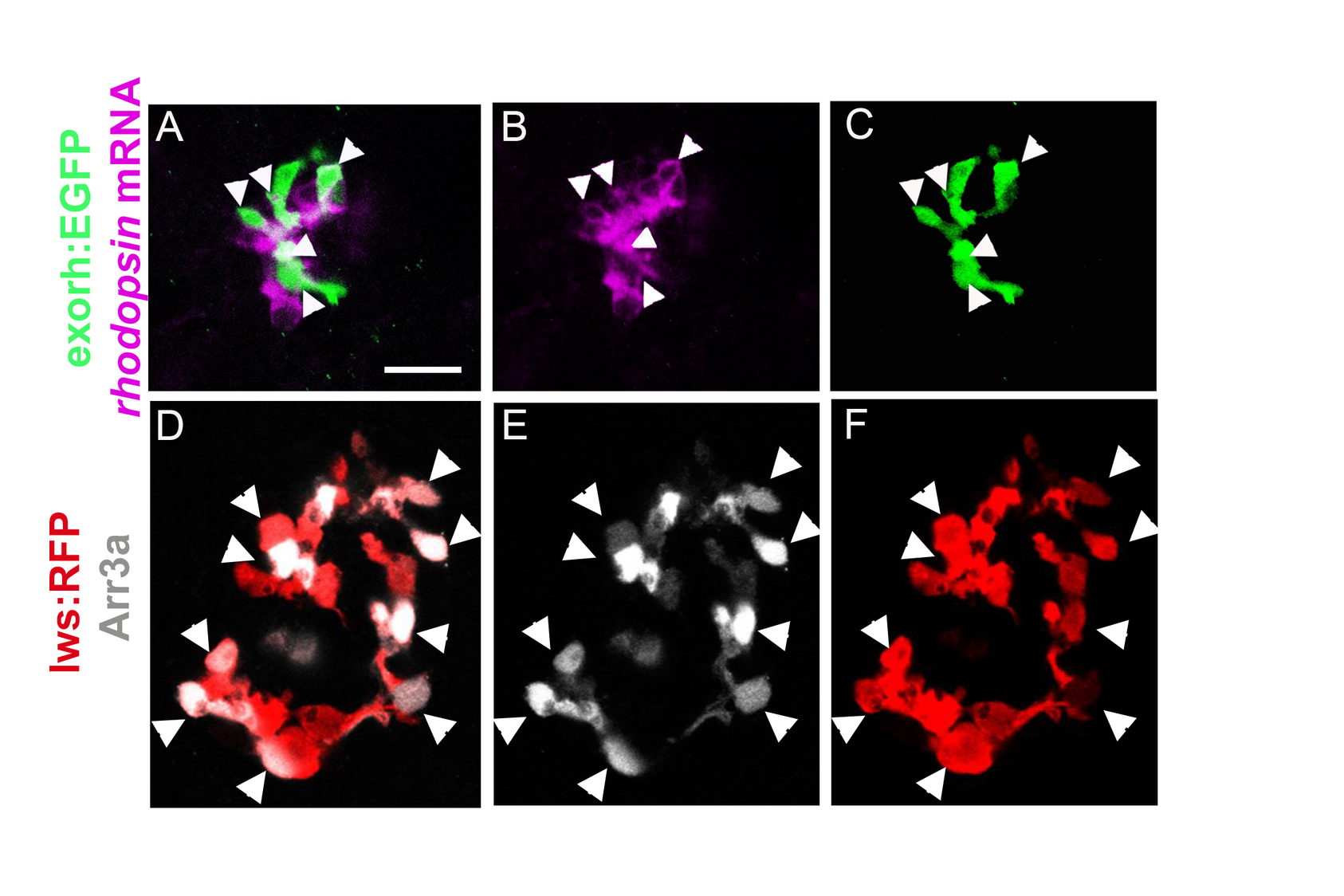

Supplement: S2 Fig — Comparison between the present description of pineal fates and previously reported “rod” and “cone” PhR fates in the pineal. (A-C) Confocal sections of 48-hpf embryos stained by in situ hybridization against rhodopsin (in magenta) and the Tg(exorh:EGFP)ja1 transgene (exorh:EGFP, in green). A high percentage of coexpression was observed between rhodopsin and the Tg(exorh:EGFP)ja1 transgene (“exorh:EGFP”; 76.5% of rho+/EGFP+ cells over the total number of EGFP+ cells, n = 11). (D-F) Confocal sections of 72-hpf embryos labeled with RFP (in red) and Arr3a (in gray) antibodies in a Tg2PAC(opn1lw1:GFP,cxxc1:RFP) transgenic line (“lws:RFP”; in red), which labels cells expressing red cone opsin [31]. All Arr3a+ cells were also RFP+ (from n = 10 embryos). White arrowheads point at double-labeled cells. Scale bar is 15 μm. Underlying data can be found in S2 Data. Arr3a, Arrestin 3a; EGFP, enhanced green fluorescent protein; exorh, exorhodopsin; hpf, hours post fertilization; RFP, red fluorescent protein. (TIF) [file pbio.2006250.s005.tif]

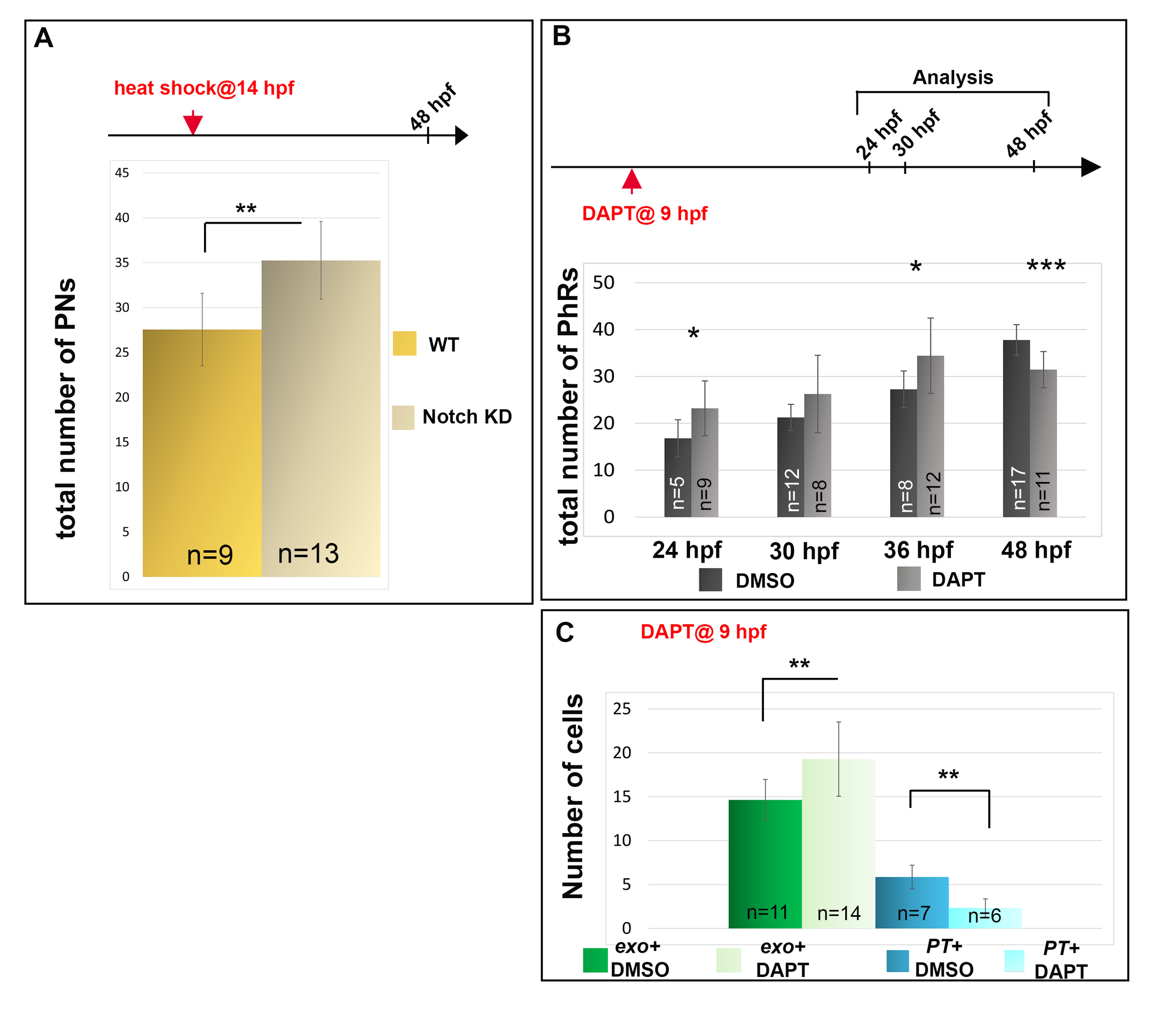

Supplement: S3 Fig — Impairing Notch activity affects the timing of PhR production and the fate of the PhRs produced. (A) Counts of HuC/D+ PN cells in Tg(hsp70l:dnXla.Rbpj-MYC)vu21 (Notch KD) and WT embryos at 48 hpf. Heat shock was performed at 14 hpf. (B) Counts of Tg(aanat2:gfp)y8+ PhRs at various stages in DAPT versus mock-treated (DMSO) embryos. Treatment was performed at 9 hpf. The stage of analysis is indicated on the x-axis. A decrease in the number of PhRs at 48 hpf is observed upon treatment with DAPT, a phenotype we previously attributed to a greater occurrence of cell death [24]. (C) Counts of exorh+ and PT+ cells in DAPT versus mock-treated (DMSO) embryos after in situ hybridization. For exorh, embryos were 48 hpf, and for PT, embryos were 54 hpf. Error bars represent SD. *p < 0.05, **p < 0.001, ***p < 0.0005 using a Mann Whitney test. Underlying data can be found in S2 Data. DAPT, N-[N-(3,5-difluorophenacetyl)-L-alanyl]-S-phenylglycine t-butyl ester; exorh, exorhodopsin; hpf, hours post fertilization; KD, knock-down; PhR, photoreceptor; PT, parietopsin; WT, wild-type. (TIF) [file pbio.2006250.s006.tif]

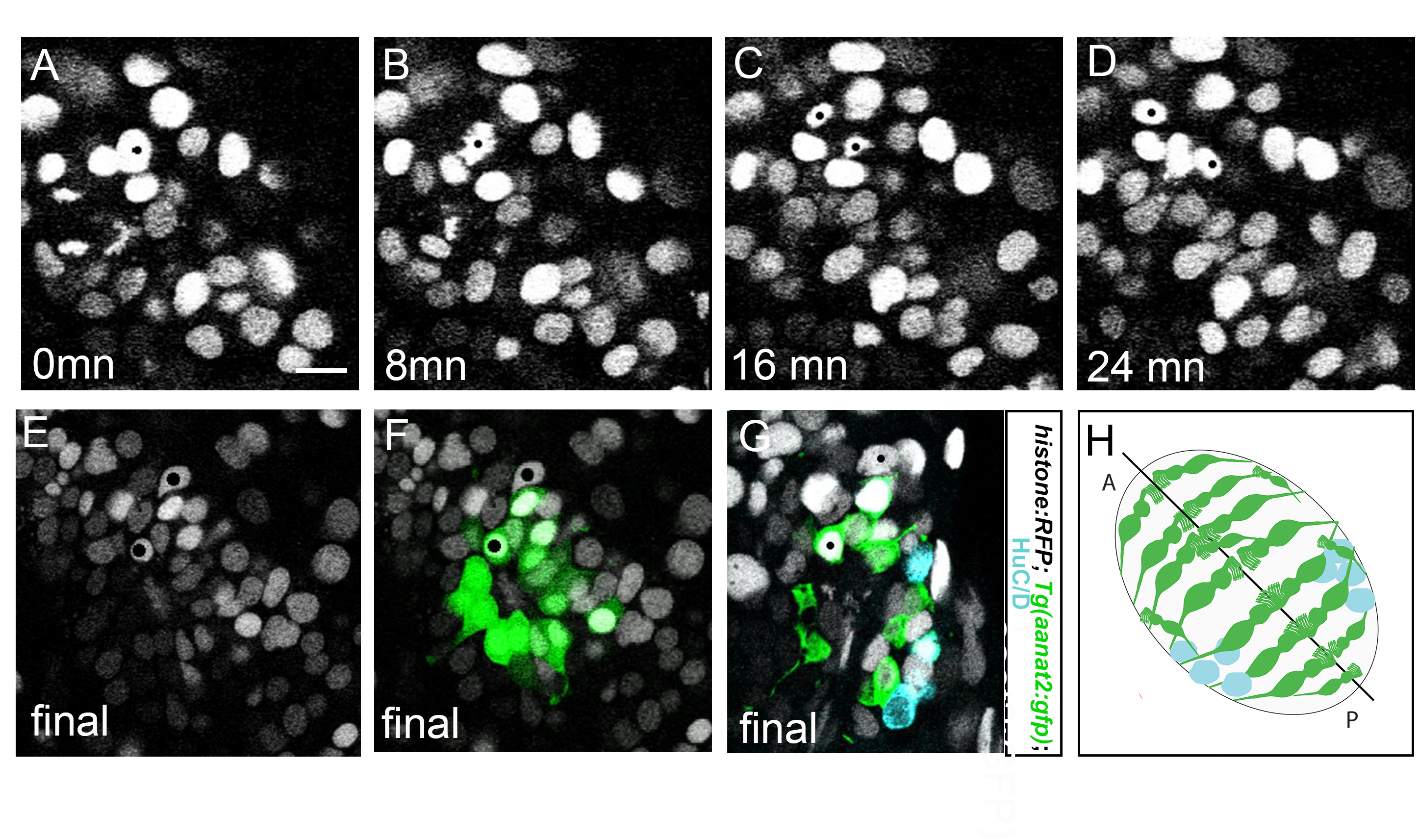

Supplement: S4 Fig — A division generating one PhR and one ø cell. (A-G) Frames from a time-lapse dataset showing a representative PhR–ø division. The mother and daughter cells are indicated with a black dot. Histone2B:RFP-labeled nuclei are in gray, whereas Tg(aanat2:gfp)y8 is shown in green, and immunostaining against HuC/D is in cyan. Anterior is toward the upper left corner. The ø cell is not a PN, as judged by the absence of HuC/D (G). (H) Schema indicating the organization and the orientation of the pineal. Anterior is toward the upper left corner, as in the individual frames. PhRs are in green and PNs in cyan. Scale bar is 15 μm. PhR, photoreceptor; PN, projector neuron. (TIF) [file pbio.2006250.s007.tif]

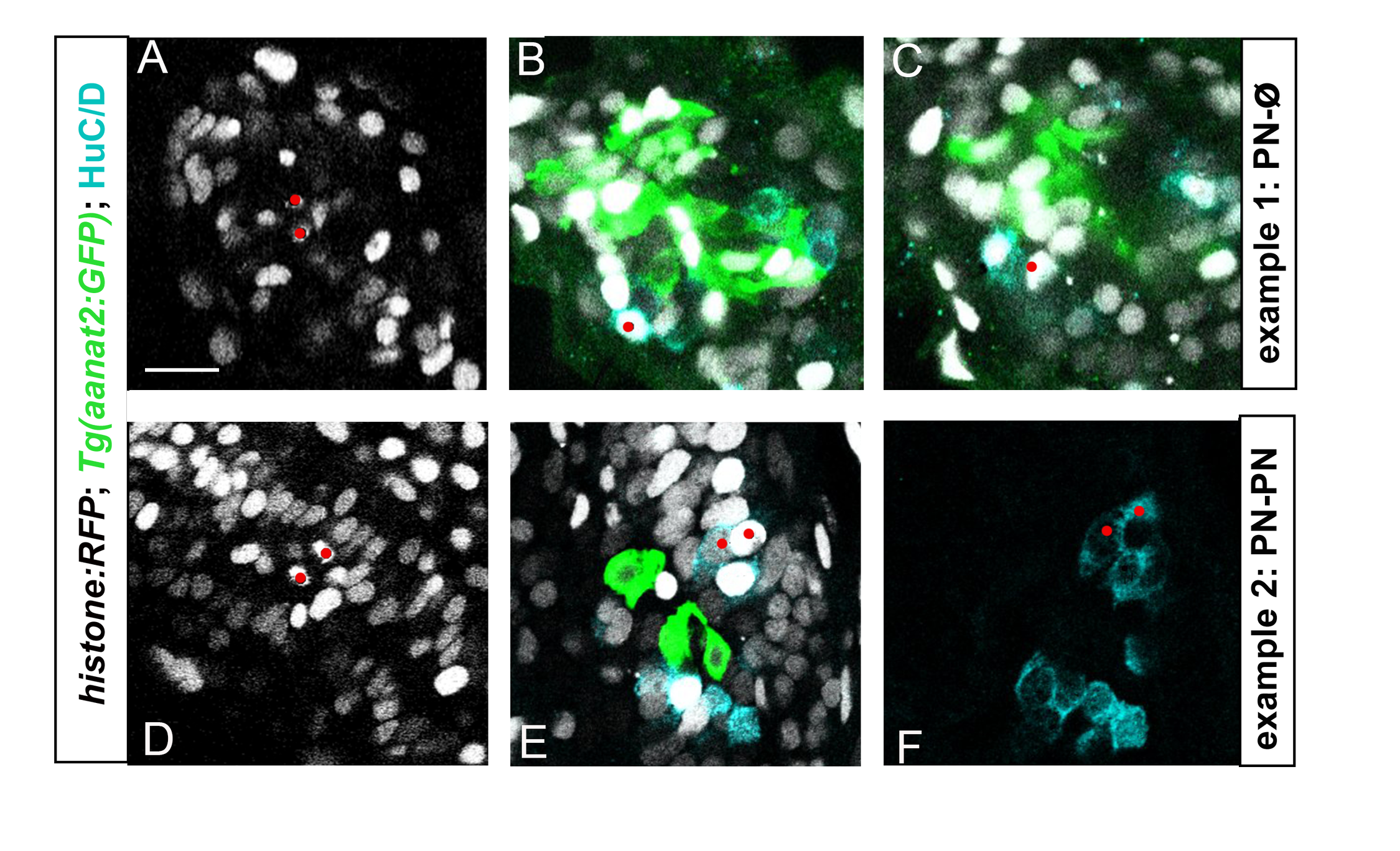

Supplement: S5 Fig — PN are born from fate-restricted progenitors. Frames from a time-lapse dataset showing examples of representative PN–ø (A-C) and PN–PN (D-F) divisions. In the case of the PN–ø division, the sister cells end up on different z planes, which are shown in B and C, respectively. The sister cells are indicated with a red dot. Histone2B:RFP+ nuclei are in gray, Tg(aanat2:gfp)y8 is shown in green, and immunostaining against HuC/D is in cyan. Anterior is toward the upper left corner. A total of 3 PN–PN and 6 PN–ø (n = 9 divisions) were successfully tracked. Scale bar is 20 μm. PhR, photoreceptor; PN, projector neuron. (TIF) [file pbio.2006250.s008.tif]

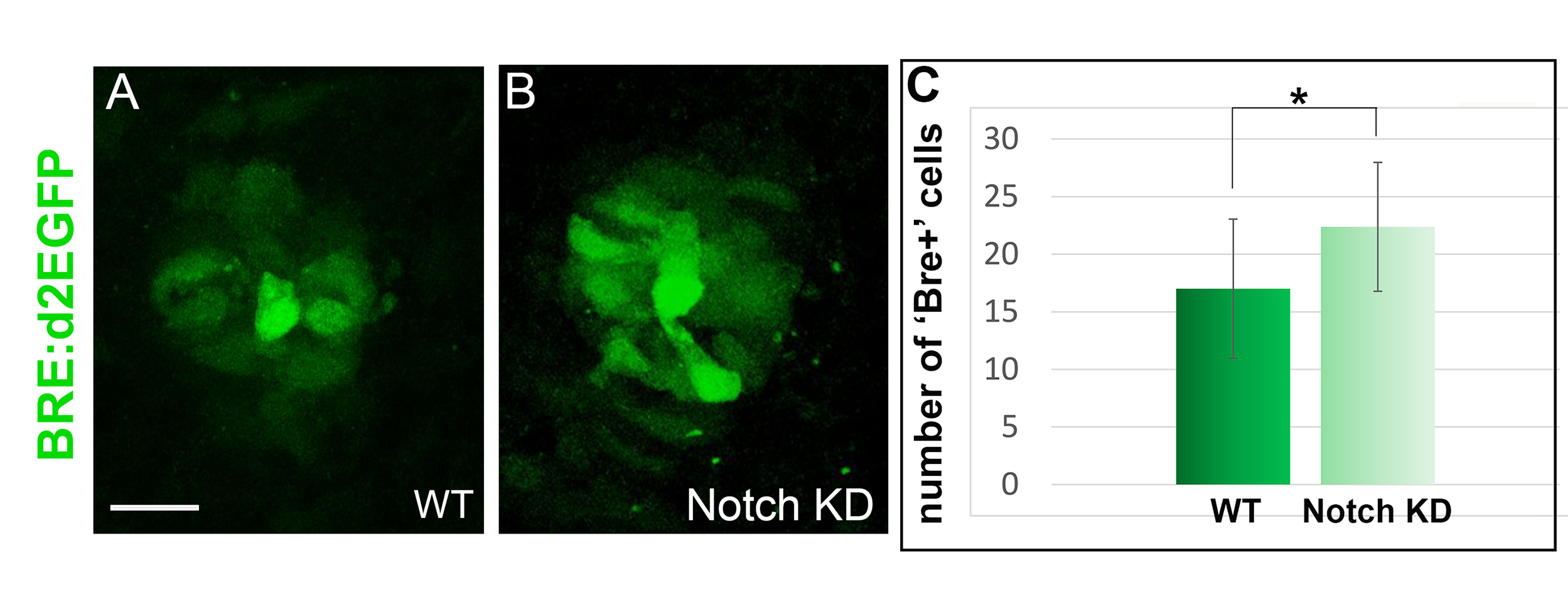

Supplement: S6 Fig — Alteration of Notch activity modifies the expression of the Tg(BMPRE-AAV.Mlp:d2EGFP)mw30 transgene. (A-B) Confocal projections of WT and Tg(hsp70l:dnXla.Rbpj-MYC)vu21 (Notch KD) embryos at 22 hpf. Embryos are shown in dorsal views. The Tg(BMPRE-AAV.Mlp:d2EGFP)mw30 transgene (BRE:d2EGFP) is shown in green. Scale bar is 15 μm. (C) Counts of dGFP+ cells in WT and Tg(hsp70l:dnXla.Rbpj-MYC)vu21 (Notch KD) embryos at 23 hpf. Underlying data can be found in S2 Data. Heat shock was performed at 14 hpf. Error bars represent SD. *p < 0.05 using a t test. Underlying data can be found in S2 Data. D2EGFP, destabilized enhanced green fluorescent protein; KD, knock-down; hpf, hours post fertilization; WT, wild-type. (TIF) [file pbio.2006250.s009.tif]
