# Supplementary material for: A Notch-mediated, temporal asymmetry in BMP pathway activation promotes photoreceptor subtype diversification
Source: PLoS Biol. 2019 Jan 31;17(1):e2006250. doi: 10.1371/journal.pbio.2006250 (PMC6372210; doi:10.1371/journal.pbio.2006250)
Supplement: Supplementary file 5 [file pbio.2006250.s003.odt]

To reconstruct movies, we used two scripts

- FILM:

open("");

titre=getTitle;

w=getWidth;

h=getHeight;

newImage("film", "8-bit Color", w, h, 1);

film=getTitle;

selectWindow(titre);

Dialog.create("adding frames");

Dialog.addMessage("Do you wish to add a frame?")

Dialog.show();

showMessageWithCancel("Do you wish to add a frame?")

run ("Run... ", "run=X ");

where X= path and folder where FILM2 is installed

- FILM2 :

selectWindow("film");

run("Add Slice");

title = "Adding frames";

msg = "move to the frame to be added and click \"OK\".";

 waitForUser(title, msg);

run("Copy");

selectWindow("film");

run("Paste");

//run("Add Slice");

showMessageWithCancel("Do you wish to add a frame?")

run("Run... ", "run=X");
